# Zinc-Releasing Fibrous Scaffolds Modulate Fibroblast, Endothelial, and Macrophage Interactions for Vascularized Tissue Engineering

**DOI:** 10.1021/acsami.5c16589

**Published:** 2026-01-06

**Authors:** Sita Shrestha, Bishnu Kumar Shrestha, Reedwan Bin Zafar Auniq, Niranjan Parajuli, Salil Desai, Narayan Bhattarai

**Affiliations:** † Department of Chemical, Biological, and Bioengineering, 3616 North Carolina A&T State University, Greensboro, North Carolina 27411, United States; ‡ Department of Nanoengineering, Joint School of Nanoscience and Nanoengineering, North Carolina A&T State University, Greensboro, North Carolina 27401, United States; § Department of Industrial and Systems Engineering, North Carolina A&T State University, Greensboro, North Carolina 27411, United States

**Keywords:** *Zinc nanoparticles*, *immunocytochemistry*, *macrophage*, *growth factors*, *wound healing*, *vascularization*, *angiogenesis*

## Abstract

Fibrous scaffolds are emerging as key biomaterials in regenerative medicine, which provide structural and biochemical support for tissue repair. Despite new advancements in the field, many fibrous scaffolds still face intrinsic limitations, including suboptimal biodegradability, inadequate bioactivity, and limited control over therapeutic release, all of which hinder their broader biomedical applications. To address these challenges, we fabricated electrospun poly­(glycolic-*co*-lactic acid) (PLGA) fibrous scaffolds embedded with various weight percentages (wt %) of zinc nanoparticles (Zn NPs), and then their physicochemical and biocompatibility properties were evaluated. The scaffold containing 1.0 wt % Zn NPs (PLZ2) exhibited controlled release of Zn^2+^, followed by a downward linear trend up to day 8. Afterward, the release rate became relatively steady over time, up to day 14, thereby avoiding burst toxicity while supporting vascularized tissue formation. The bioactivity of these scaffolds was systematically evaluated using multiple cell types, such as human dermal fibroblasts (HDFn), human umbilical vein endothelial cells (HUVECs), and RAW264.7 macrophages, showing important properties in wound healing and angiogenesis. Further, indirect coculture of HUVECs and HDFn was studied to assess cell–to–cell interactions. The controlled release of Zn^2+^ from PLZ2 promoted fibroblast-to-myofibroblast differentiation significantly, indicated by increased vimentin and α-smooth muscle actin expression and enhanced secretion of angiogenic growth factors–vascular endothelial growth factor (1.34-fold) and basic fibroblast growth factor (1.47-fold) at day 7. These soluble growth factors stimulated HUVECs’ survival, enhanced their migration, and promoted the formation of capillary-like networks. Simultaneously, scaffolds promoted the transformation of macrophages into M1 and M2 phenotypes, identified through the immunostaining of macrophage polarization markers, inducible nitric oxide synthase (iNOS) and Arg1, and further confirmed by Western blot analysis. In addition, endothelial functionality was further supported by CD31 and VE-cadherin upregulation. These findings indicate that the indirect coculture system in conditioned culture medium with Zn^2+^ effectively stimulates HUVECs and may also influence other cell types to generate capillary-like structures. This approach holds promise for tissue regeneration and applications where vascularization is essential.

## Graphical abstract

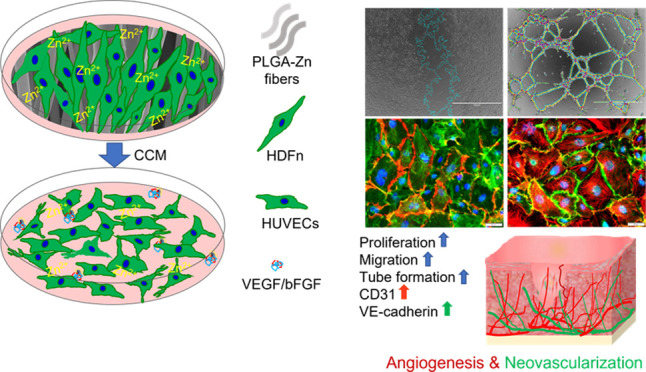

## 1 Introduction

Tissue vascularization is a fundamental metabolic process that delivers oxygen and nutrients and removes metabolic waste.^1^ Achieving sufficient vascularization within engineered tissue constructs remains a major challenge due to limitations such as inadequate porosity, poor scalability, low cell viability, and the complexity of tissue geometries.^2^ In vitro, replication of vascularization enhances cell–cell communication and cell-extracellular matrix (ECM) interactions, which are important for tissue fusion and successful integration with the host tissue. These interactions play key roles in regulating cellular functions such as proliferation, migration, differentiation, and the formation of vascular networks.^3^ The formation of new blood vessels or neoangiogenesis is a complex process that is necessary in wound healing and tissue regeneration.^4^ In tissue engineering, coculturing multiple cell types within regenerative bioscaffolds can provide diverse cell sources with multipotent differentiation capacity. This approach promotes tissue homeostasis, supports cell metabolism, and initiates angiogenesis, ultimately facilitating wound repair.^5^ Notably, the coculture systems enhance neovascularization through paracrine signaling, as evidenced by the upregulation of angiogenic factors such as vascular endothelial growth factor (VEGF) and basic fibroblast growth factor (bFGF).^6^

Proangiogenic regenerative biomaterials offer a viable alternative to recombinant growth factors by promoting endothelial cell growth, differentiation, and tubule formation.^7,8^ Coculture system accelerates cellular activity and vascular network formation. Montesano et al. showed that fibroblasts stimulate endothelial angiogenesis via paracrine signaling, forming capillary-like tubes.^9^ Indirect coculture of fibroblasts and endothelial cells enhances the secretion of bFGF and VEGF, improving endothelial proliferation and tubulogenesis.^8^ Angiogenesis also depends on adhesion molecules like VE-cadherin and CD31, which maintain vascular integrity and regulate cell signaling.^10^ The coculture of hUVECs in calcium phosphate-based scaffolds with various stem cells, such as hUCMSCs, hBMSCs, hiPSC-MSCs, and hESC-MSCs, increases the expression of CD31, indicating enhanced angiogenic capacity.^11^ Cocultures also support endothelial migration and network formation, facilitated by VE-cadherin and growth factors.^11^ In one study by Levorson et al., indirect coculture of MSCs and chondrocytes on polymer scaffolds led to the greater deposition of ECM components, supporting the advantages of coculture in tissue regeneration.^12^

Biodegradable metallic particles, particularly those of Zn, magnesium (Mg), and iron (Fe), have shown strong potential in supporting tissue repair and preventing infection.^13^ These particles act as biocatalysts, influencing gene expression, protein synthesis, and signal transduction.^14^ Metals like Mg, Zn, and their alloys are widely explored for biomedical implants such as bone fixation devices, stents, and surgical pins.^15^ Upon degradation, they release metal ions (e.g., Zn^2+^ and Mg^2+^) and hydrogen, both of which offer antioxidant, anti-inflammatory, and tissue-regenerative effects.^16^ However, uncontrolled accumulation of these ions can lead to cytotoxicity; excess Zn^2+^, for example, can disrupt growth factor signaling and trigger apoptosis.^17^ Controlled-release systems are, therefore, essential to harness their benefits while minimizing toxicity. Our lab and others have created such controlled-release systems that embed these particles in biopolymers or proteins, forming microspheres, fibrous mats, foams, and 3D-printed scaffolds.^18,19^ For instance, controlled Zn^2+^ release (∼27.5 nM in 3 days) from a PCL-based fibrous scaffold enhanced NIH/3T3 fibroblast proliferation.^20^ Likewise, a Zn-based metal–organic framework such as PgC_3_Zn MOF demonstrated antioxidant, antibacterial, and anti-inflammatory properties, promoting fibroblast growth, angiogenesis, and collagen deposition, key factors in wound healing.^21^ Indeed, the pH of the cellular environment greatly influences the long-term stability of the MOF, for example, by causing the uncontrolled accumulation of metal particles due to rapid dissolution and increasing oxidative stress. This stress affects fibroblast proliferation and limits the re-epithelialization of keratinocytes. Moreover, the MOF of PLGA-based nano/micro structures has been employed in a wide range of biomedical applications, including grafts, scaffolds, implants, and sutures, owing to their excellent biodegradability and biocompatibility.^22^ The weak mechanical strength and hydrophobic nature further restrict PLGA in designing on-demand therapeutic devices.^23^ To overcome these challenges, researchers have incorporated metallic and ceramic nanoparticles into PLGA-based fibrous scaffolds. These nanostructured constructs closely mimic the physical architecture of the ECM, enhancing the scaffold’s functional properties and promoting tissue repair and regeneration.^24^

In this study, we investigated the effects of controlled Zn^2+^ release from PLGA fibers on the vascularization. PLGA fibrous scaffolds embedded with varying wt % of Zn NPs were fabricated via electrospinning, followed by characterization of their physicochemical properties and biocompatibility. The bioactivity of these scaffolds was systematically assessed using multiple cell types, including HDFn, human umbilical vein endothelial cells (HUVECs), and RAW264.7 macrophages. We hypothesize that HDFn, cultured on Zn NPs containing fibrous scaffolds, can enhance the activity of HUVECs and promote angiogenesis and vascularization through paracrine signaling in an indirect coculture system. The conditioned medium collected from HDFn cultured on Zn-containing scaffolds is expected to stimulate neovascularization in HUVECs via paracrine mechanisms. This approach encourages and ensures tissue regeneration in a coculture system, where the platform exhibits a cellular microenvironment enriched with Zn as cofactors in cellular metabolism. The controlled and sustained release of Zn^2+^ from fibrous scaffolds induces HDFn to secrete soluble growth factors, enhancing their growth, proliferation, and differentiation and supporting endothelial cell function and vascular tissue formation. This study provides valuable insights into how Zn^2+^ modulates both monoculture and coculture environments by influencing cell–cell interactions and micropolarization, suggesting a promising strategy for wound healing and tissue regeneration. Scheme 1 presents a schematic illustration of the experimental design, highlighting the indirect coculture microenvironment and the proposed role of Zn^2+^ in simulating cutaneous-like wound healing and regenerative processes.

**Scheme 1:**
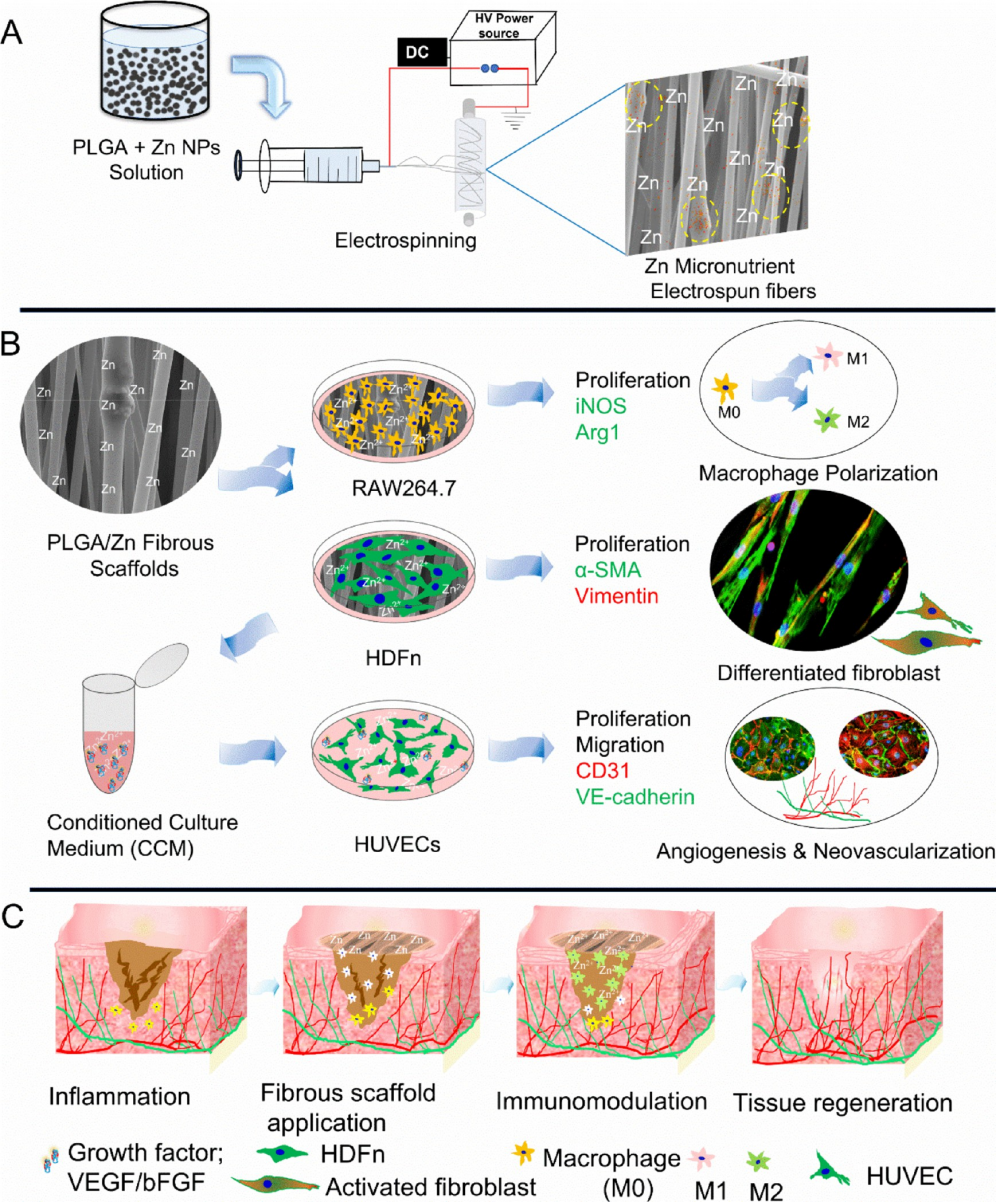
Schematic Illustration of Experimental Design: (A) Electrospinning Setup Utilized to Fabricate Zn Nanoparticles Incorporated Fibrous Scaffolds; (B) Establishment of an Indirect co-Culture Microenvironment with Multiple Cell Types; and (C) Simulation of Cutaneous Wound Healing and Tissue Regeneration Process

## 2 Experimental Section

### 2.1 Materials and Reagents

Poly­(D, l-lactide-*co*-glycolide) 75:25 (PLGA, Resomer RG 755 S) was purchased from Evonik Operations GmbH, Kirschenallee, Darmstadt, Germany. Zinc nanoparticles (Zn NPs), 3-(4,5-Dimethyl-2-thiazolyl)-2,5-diphenyl-2H tetrazolium bromide (MTT, methylthiazolyldiphenyl-tetrazolium bromide) assay kit, heparin sodium salt (H3393), and bovine serum albumin (BSA) were purchased from Sigma-Aldrich, St. Louis, MO, USA. Trifluoroethanol (TFE), endothelial cell growth supplement (ECGS; CB-40006), trypsin–EDTA (Gibco), AlamarBlueTM cell viability reagent (AB), paraformaldehyde (PFA), phosphate buffer saline (PBS) (Life Technologies, Carlsbad, CA, USA), Triton X-100, Actin GreenTM 488 ReadyprobesTM reagent (Invitrogen), 4′6, diamidino-2-phenylindole dihydrochloride (DAPI, Invitrogen), were obtained from Thermo Fisher Scientific (Waltham, MA, USA). Primary dermal fibroblast normal; human, neonatal (HDFn, PCS, 201-010TM), fibroblast basal medium (PCS, 201–030), fibroblast growth kit-serum free (PCS, 201–040), streptomycin-amphotericin B solution (PCS, 999–002), trypsin–EDTA for primary cells (PCS, 999–003) and trypsin neutralizing solution (PCS, 999–004), HUVEC (CRL-1730TM), F–12K medium (30–2004), and fetal bovine serum (FBS, 30–2020) were purchased from ATCC (Manassas, VA, USA).

### 2.2 Preparation of Fibrous Scaffolds

The fibrous scaffolds were prepared using an in-house electrospinning setup.^25^ Briefly, the electrospinning suspension was prepared by dispersing Zn NPs at different weight percentages (0.5, 1.0, and 2.0%) into TFE. Then, PLGA (25 wt %) was added to it and stirred overnight in an airtight glass bottle using a magnet. The homogeneous PLGA/Zn mixture was loaded in a 10 mL syringe with an attached 18 gauge diameter hypodermic needle for electrospinning. The flow rate was set at 1.0 mL/h, and the distance between the tip and collector was maintained at approximately 12 cm with an applied 15 kV. The grounded rotating drum of the collector was wrapped with aluminum foil to collect the fibers. The fibrous scaffolds were henceforth referred to as PLZ0 for the fibers of PLGA only and PLZ1, PLZ2, and PLZ3 corresponding to 0.5, 1.0, and 2.0 wt % of Zn NPs by dry weight of PLGA, respectively. The fibers were completely dried before further analysis and characterization.

### 2.3 Characterization of Physicochemical Properties

Scanning electron microscopy (SEM, Jeol-800) equipped with energy-dispersive X-ray spectroscopy (EDS) (Quantax 70, Bruker Corporation, Billerica, MA, USA) was used to observe the surface morphology of the fibers. Scaffolds were prepared by cutting them into small pieces, attaching them to carbon tape, and sputter-coating with gold–palladium using a coating device (Leica EM ACE200, IL, USA) for 30s (coating depth = 5 nm) at 15 mA. SEM images were taken at an accelerating voltage of 15 kV. EDS analysis was conducted to confirm the Zn NPs in the fibrous membranes. Fiber diameter distribution was assessed by using SEM images and analyzed with ImageJ software. Measurements were taken from 50 individual fibers per group (*n* = 3), and a frequency distribution histogram was generated. Furthermore, high-resolution transmission electron microscopy (HRTEM; JEOL-2100Plus) was employed to confirm the incorporation of Zn NPs within the fibers. Briefly, the fibers were transferred to the TEM grid through quick ultrasonication in absolute ethanol and operated at an accelerating voltage of 80 kV to observe the fibers. In addition, the crystallinity of each material was also examined using X-ray diffraction (Rigaku MiniFlex XRD with D/teX Ultra2 detector, Wilmington, MA) with a 2θ range of (5–60) ° at a scan rate of 10°/min. The Fourier transform infrared (FTIR) spectra were recorded by an FTIR spectrometer (Agilent 670, Sant Clara, CA, USA). TA Q200 differential scanning calorimetry (DSC) analysis was performed to evaluate the temperature and heat flow of the different fibers. Thermogravimetry analysis (TGA) was conducted to observe the mass loss and was measured using a TGA instrument (TGA 5500, USA). The mechanical properties of the fiber (customized template constructed from cardstock; 50 × 35 mm) were examined using a TA. XT Plus Texture Analyzer (Hamilton, MA). The hydrophilicity of the fibrous scaffolds was determined through the static contact angle measurement using the sessile drop method (KRUSS drop shape analyzer, DSA25E, Germany) at room temperature (RT). Details of the procedure and results of physicochemical properties characterization are presented in the Supporting Information.

### 2.4 In Vitro Assessment of Zn^2+^ Release and Degradation of Fibrous Scaffold

The release of Zn^2+^ from the scaffolds was analyzed to evaluate their potential cytotoxicity in vitro. Each scaffold (*n* = 3) with equal diameter was prepared on glass coverslips and fixed at the bottom of 48-well plates. The scaffolds were sterilized, followed by washing with 1× PBS and soaking in cell culture media with and without seeding HDFn cells (see details of cell culture in the following section: cell culture study). At predetermined time points (1, 2, 3, 5, 8, 11, and 14 days), the cell culture media were collected, and the Zn^2+^ concentration was measured using the Zn Quantification Kit (catalog ab102507, Abcam Inc., Waltham, MA, USA). The amount of Zn^2+^ released was quantified under cell-culture conditions using a colorimetric assay at 560 nm. Further, an enzymatic biodegradation test was performed with these scaffolds. The dry scaffolds were immersed in PBS containing 10 mg/mL lysozyme (Millipore Aldrich Chemie GmbH, Kappelweg 1, 91625 Schnelldorf, Israel) for 1, 3, and 7 days. The concentration of the enzyme was based on the ASTM standard (F-1635-95). After various time intervals, the scaffolds were washed with DI water several times and lyophilized. SEM was used to examine the morphologies of lyophilized scaffolds. Details of the procedure and results are presented in the Supporting Information.

### 2.5 Cell Culture

Primary HDFn were cultured in fibroblast basal medium with fibroblast growth kit-serum free, containing each of the following growth supplements: l-glutamine, hydrocortisone hemisuccinate, HLL supplement (Human serum albumin, linoleic acid, lecithin), rh FGF-β, rh EGF/TGF-β1 supplement, recombinant human insulin, and ascorbic acid, along with streptomycin-amphotericin B solution (dilution 1:1000) at 37 °C with 5% carbon dioxide (CO_2_) in a 95% humidified atmosphere. After 90% confluency, the cells were passaged by dissociation with trypsin–EDTA for primary cells and a trypsin neutralizing solution. Fibroblasts were used in passage three in this study. HUVECs were cultured in F–12K medium and heparin sodium salt solution supplemented with 10% FBS and ECGS under cell culture conditions. After confluency, cells were harvested using trypsin–EDTA. The cells were used in passage eight for this study.

Further, macrophages (RAW264.7, ATCC Cell Line Bank 1658, Manassas, VA, USA) were cultured in DMEM supplemented with 10% FBS and 1% penicillin–streptomycin (P/S) at 37 °C with 5% CO_2_ in a humidified atmosphere until 90% confluence was reached. Raw264.7 was used for each experiment at passages 6–8. The cell culture medium was replenished every second day of the culture period.

### 2.6 Cell Viability and Proliferation Assays

The scaffolds (*n* = 3) were prepared on circular glass coverslips (12 mm diameter) and fixed at the bottom of 48-well plates (Thermo Fisher Scientific, USA). Before cell seeding, the prepared scaffolds were sterilized under UV light for 3 h and washed with 70% ethanol, followed by PBS (1X). After pretreating the scaffolds in the medium for 1 h, HDFn and HUVECs at a density of 1.5 × 10^4^/well were seeded separately at the center of the scaffolds. The cells were replenished with fresh culture medium every 2 days. The biocompatibility of the fabricated scaffolds and proliferation were assessed using the MTT assay. Absorbance was measured at 540 nm by using a microplate reader. Furthermore, the biocompatibility and viability of HDFn and HUVECs on the scaffolds were separately assessed using the AB assay. The cell seeding was performed similarly to that of the MTT assay described earlier. A microplate reader (BMG Labtech Microplate, Cary, NC, USA) was used to measure the absorbance at 570 nm, with 600 nm as a reference.

### 2.7 Live/Dead Assay, Attachment, and Morphological Study of HDFn and HUVECs

Cell survivability and spreading on the scaffolds were assessed using a live/dead assay kit (PerkinElmer LLC, Via AOPI Staining Solution; Fisher Scientific, USA). HDFn and HUVECs were cultured on various scaffolds for 5 days, as described above (cell viability and proliferation assays). Cells were visualized using a fluorescence microscope (Olympus IX83 microscope with the Olympus cellSens Dimension software, Olympus Corporation, Shinjuku, Tokyo, Japan). Fluorescence images (*n* = 3) were used for the quantification of live (green) and dead (red) cells on each scaffold using ImageJ software (J 1.53c, NIH, Bethesda, MD, USA).

The morphology, growth, and attachment of HDFn and HUVECs on different scaffolds were evaluated in vitro using a fluorescence microscope after 1 and 3 days of incubation at 37 °C. For fluorescence imaging, the cells at a density of 1.0 × 10^4^/well were separately seeded onto scaffolds of similar size to those used in the viability test. After the predetermined culturing period, the cells were washed with PBS and fixed with 4% PFA solution for 10 min. The cells were permeabilized with 0.2% Triton X-100 for 3 min at RT and blocked with 1% BSA for 30 min. The cells were stained with Actin Green 488 ReadyProbes reagent for the cytoplasm for 20 min and DAPI for nuclei for 5 min in the dark. The images were captured using an Olympus IX83 microscope. Fluorescence intensity (*n* = 3) was quantified using ImageJ software.

### 2.8 Immunocytochemistry Assay for HDFn

To assess the differentiation of HDFn cultured with different scaffolds, ICC staining was performed according to the company’s procedure. Briefly, HDFn cultured for 7 and 14 days were washed with PBS and fixed with 4% PFA. The cells were then permeabilized with 0.2% Triton X-100 for 5 min at RT. After rinsing with PBS-T, nonspecific binding sites were blocked with 1% BSA for 30 min. Next, the cells were incubated overnight at 4 °C with primary antibodies: antivimentin (ab8978, Abcam, USA) and antialpha-smooth muscle actin (α-SMA, ab124964, Abcam). After washing with PBS-T, the cells were incubated with secondary antibodies: goat antirabbit IgG H and L (Alexa Fluor 488; ab150077, Abcam) and goat antimouse IgG H and L (Alexa Fluor 594; ab150116, Abcam) for 1 h at RT in the dark. The nuclei were counterstained with DAPI for 10 min. Fluorescence images of the cells were taken using an Olympus IX83 microscope. The intensity of vimentin and α-SMA was quantified by analyzing the fluorescence images using ImageJ software.

### 2.9 Measurement of bFGF and VEGF Secreted by HDFn

The secretion of soluble growth factors, VEGF and bFGF, from HDFn was measured using enzyme-linked immunosorbent assay (ELISA) according to the company’s protocol (Human VEGF ELISA Kit, ab222510, Abcam, USA; bFGF ELISA Kit, HU9915, Biotechnology Systems, USA). Briefly, about 200 μL of cell culture supernatants from in vitro culture of HDFn seeded on fibrous scaffolds was collected after 1, 3, 5, and 7 days. Then, standards and supernatants (50 μL for VEGF and 100 μL for bFGF) were added to the wells of a plate coated with capture antibodies. After being incubated for 1 h at RT (for VEGF) and 40 min at 37 °C (for bFGF), the plates were washed with the provided wash buffer. For VEGF, 100 μL of TMB development solution was added to each well, and the mixture was incubated for 15 min in the dark on a plate shaker. Following incubation, a stop solution was added, and the optical density was measured at 450 nm. For bFGF, 50 μL of distilled water and 50 μL of biotinylated antibody were added to each well (except the blank), and the plate was incubated at 37 °C for 20 min. After washing, 100 μL of the enzyme conjugate was added, and the plate was incubated at 37 °C for 10 min. Then, 100 μL of the substrate solution was added and incubated at 37 °C for 15 min in the dark. Absorbance was measured at 450 nm within 30 min after addition of the stop solution.

### 2.10 Macrophage Cell Proliferation and Polarization Study

To evaluate the scaffolds with RAW264.7 cells, preparation and sterilization were conducted as described above (cell viability and proliferation assays). The proliferation and viability of RAW264.7 cells were also assessed using an MTT assay and an AB assay. The effect of Zn^2+^ on macrophage phenotype was evaluated through the analysis of macrophage polarization protein markers, including inducible nitric oxide synthase (iNOS) and liver arginase (Arg1). In this experiment, RAW264.7 cells were treated with 0.01 μg mL^–1^ lipopolysaccharide (LPS) for 6 h to induce polarization. RAW264.7 cells were seeded at a density of 1.5 × 10^4^/well, pipetted at the center of the scaffolds, and incubated at 37 °C for 3 days to induce M1 and M2 macrophage polarization. After 3 days, the cells were fixed, permeabilized, rinsed, and blocked with BSA. Next, the cells were incubated overnight at 4 °C with primary antibodies: anti-iNOS (ab178945, Abcam) and antiliver arginase (ab96183, Abcam). After washing, the cells were incubated with a secondary antibody, goat antirabbit IgG H and L (Alexa Fluor 488; ab150077, Abcam). The nuclei were counterstained with DAPI. Images were taken using the Olympus Microscope. The intensity of the macrophage polarization protein markers was quantified by ImageJ software.

### 2.11 HDFn-HUVEC Indirect Contact Coculture Method

In the HDFn-HUVEC indirect contact coculture method, cell culture media were collected from HDFn cultured on different scaffolds. Briefly, HDFn were seeded onto various scaffolds in 48-well plates at a density of 3 × 10^4^/well in complete media. After 24 h, the medium was collected, termed the conditioned culture medium (CCM), and stored at 4 °C. Concurrently, HUVECs were cultured on glass coverslips placed in 48-well plates without scaffolds, starting on the same day as the HDFn culture. After 24 h, the collected CCM from HDFn was added to the HUVECs attached to the glass coverslip. Fresh media (FM) were used as controls for each cell culture study. The metabolic activity of the cells was assessed using the MTT and AB assays after 1, 3, and 5 days. Further, a live/dead assay was also performed to examine the cell survivability on the CCM.

### 2.12 Vascular Differentiation of HUVECs

#### 2.12.1 In Vitro Scratch Closure Assay

An in vitro scratch wound assay with HUVEC cultures was performed to evaluate the wound closure rate. Briefly, HUVECs were seeded at a density of 1.5 × 10^5^/well in 24-well plates and allowed to form a confluent monolayer. A wound was created by scratching the culture surface with a pipet tip, leaving a cell-free strip. This generated a gap that mimicked a wound and allowed the remaining cells to migrate and close the wound. The wound diameter variation across the three experiments was determined and normalized. After scratching, the cells were washed with 1× PBS to remove any detached cells or fragments before incubation with media extracted from different scaffolds (PLZ0, PLZ1, and PLZ2) and CCM from HDFn. Freshly prepared media (FM) served as a control for monoculture and conditioned media (CM) from HDFn without a scaffold were used as a control for the coculture. The wound area and cell migration were monitored at 0, 12, and 24 h using a phase contrast inverted microscope. The percentage of the cell-free area covered by migrating cells was quantified by using the wound healing size tool in ImageJ software.

#### 2.12.2 In Vitro Tube Formation Assay

To investigate the angiogenic properties, the effect of Zn on monoculture and the influence of CCM in the indirect coculture of HUVECs were examined using a tube formation assay. Briefly, 24-well plates were coated with ice-cold Matrigel Matrix Basement Membrane (ref- 354234, Corning, USA) and incubated at 37 °C for 1 h to allow the Matrigel to solidify. HUVECs were then harvested from the culture plates and seeded at a density of 1.5 × 10^5^/well onto the Matrigel layer. The cells were incubated at 37 °C with media extracted from different scaffolds and various CCMs. FM served as a control for monoculture, and CM was used as a control for the coculture. Images of the tubular structures were captured using an inverted microscope at 3 and 6 h. Data quantification was performed using the Angiogenesis Analyzer with ImageJ software.

#### 2.12.3 ICC Assay of HUVECs

To assess protein expression related to endothelial cell differentiation, VE-cadherin immunostaining and CD31, markers for cell adhesion and vascular differentiation, were performed. Briefly, HUVECs were seeded in 48-well plates and cultured for 7 days with media extracted from different scaffolds and CCM. FM was used as a control for monoculture, and CM was used as a control for the coculture. Cells were then fixed, washed, and permeabilized with Triton-X100 and blocked with BSA. The cells were incubated overnight at 4 °C with primary antibodies: VE-cadherin Polyclonal Antibody (PA5-19612, Thermo Fisher Scientific, Invitrogen) and anti-CD31 antibody (ab24590, Abcam). Afterward, the cells were incubated at RT for 1 h with secondary antibodies: goat antirabbit IgG H and L (Alexa Fluor 488; ab150077, Abcam) and goat antimouse IgG H and L (Alexa Fluor 594; ab150116, Abcam). Nuclear DNA was labeled with DAPI. F-actin was stained with ActinGreen 488 ReadyProbes Reagent (Alexa Fluor 488 phalloidin) and ActinRed 555 ReadyProbes Reagent (Rhodamine phalloidin). Images were taken with an Olympus IX83 microscope.

### 2.13 Western Blot Assay

The expression level of proteins was evaluated by conducting a Western blot assay. Briefly, RAW264.7 cells at 1 × 10^6^/well and HDFn at 5 × 10^6^/well were seeded on the scaffolds for days 3 and 7, respectively. The HUVECs at 1 × 10^5^/well were seeded with CCM in 6-well plates for 7 days. The proteins extracted from the cells were quantified using the Bradford protein assay (Bio-Rad, Richmond, CA), loaded onto a 10% SDS-polyacrylamide gel, and separated by electrophoresis. The polyacrylamide gel with separated proteins was transferred onto nitrocellulose membranes and blocked with 5% BSA for nonspecific proteins. Subsequently, the membranes were incubated overnight at 4 °C with primary antibodies against iNOS, TNF-α (ab66579; Abcam), Arg1, Type III collagen (PA5-34787; Invitrogen), α-SMA, VEGF (MA5-13182; Invitrogen), CD31, VE-cadherin, and β-actin (AM4302; Invitrogen). Immunoreactivity was determined using horseradish-peroxidase-conjugated secondary antibodies at RT for 1 h. Finally, protein signals were detected with an enhanced chemiluminescence solution and scanned under an iBright 1500 microscope (Invitrogen). Band intensities were quantified by densitometric analysis using iBright Analysis software and normalized to β-actin.

### 2.14 Statistical Analysis

OriginPro software version 2023 (Origin Lab, Northampton, MA, USA) was used to plot the statistical data. Microsoft Excel was used to analyze all statistical quantitative data, presented as the mean ± standard deviation (SD, *n* = 3). A one-way analysis of variance (ANOVA) test was performed, followed by Tukey’s post hoc analysis to determine statistically significant differences between the groups. The α-value was set to 0.05, and significance was considered at p-values of *p < 0.05, **p < 0.01, and ***p < 0.001.

## 3 Results and Discussion

### 3.1 Physiochemical Properties of Fibrous Scaffolds

#### 3.1.1 Surface Morphology

The surface morphology of the fibrous scaffolds and the fiber diameter frequency distribution histogram are shown in Figure 1A–C. The SEM image of the PLZ0 revealed an interwoven fibrous network with most fiber diameters ranging from 1100 to 1700 nm, as indicated by the frequency distribution analysis (Figure 1A). The PLZ0 exhibited smooth-surfaced fibers with a more aligned structure and an average fiber diameter of 1501 nm. In comparison, slightly larger and denser fibers were observed on PLZ1 (Figure 1B). The incorporation of 0.5 wt % Zn NPs results in the formation of continuous fibers, with a diameter distribution ranging from 1500 to 2400 nm and an average diameter of 1890 nm. Notably, the fiber diameters in PLZ1 appeared to be relatively larger in the frequency distribution compared to those in PLZ0 and PLZ2 (Figure 1. Importantly, the addition of Zn NPs did not alter fiber morphology significantly, suggesting that the particles were uniformly embedded and well-distributed. However, PLZ2 exhibited a more aligned morphology with smaller fiber diameters. The majority of fibers were in the range of 900 to 1400 nm in diameter, with an average diameter of 1190 nm. The SEM images of PLZ2 (Figure 1C) also showed beads due to the encapsulation of Zn NPs within the fibers.

**Figure 1:**
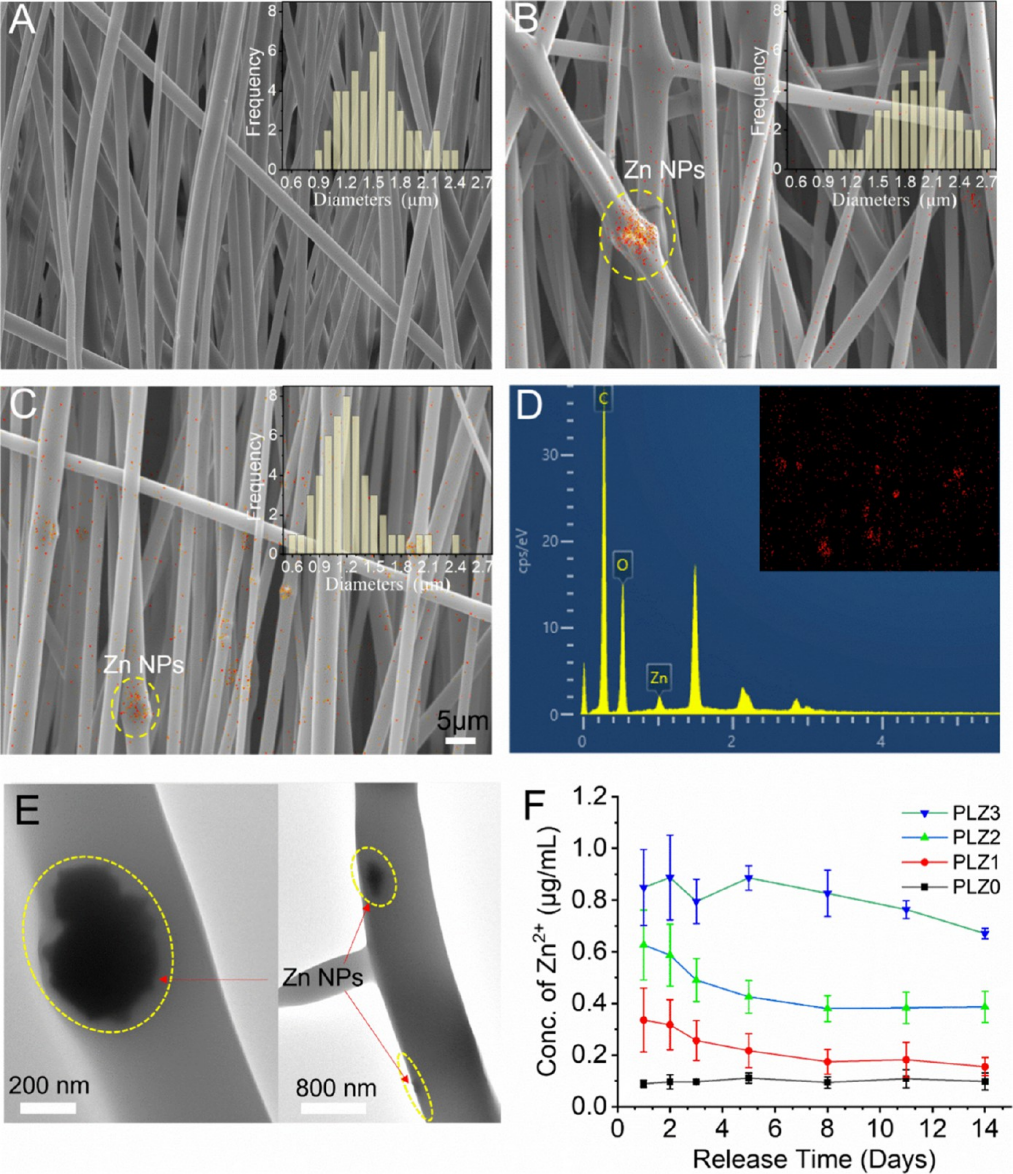
Surface morphology and diameter distributions of fibrous scaffolds. SEM-EDS micrographs show the morphology of the fibers at different compositions: (A) PLZ0, (B) PLZ1, and (C) PLZ2. Scale bars = 5 μm. Insets show the frequency distribution of fiber size corresponding to the diameters of the fibers (*n* = 50). (D) SEM-EDS elemental mapping of PLZ2 fibers confirms the presence of Zn NPs (red spots in the inset represent the Zn NP distribution). (E) HR-TEM images of PLZ2 fibers (scale bar = 200 and 800 nm, left and right, respectively). Quantitative analysis of Zn^2+^ released from the fibrous scaffolds. (F) Representative release profiles of Zn from the scaffolds, in vitro cell culture conditions at different periods (*n* = 3).

Typically, the presence of metallic nanoparticles enhances conductivity, leading to the formation of finer fibers.^26^ We observed this trend when the Zn NPs increased beyond 1.0 wt %. However, blending 2.0 wt % Zn NPs with the PLGA (i.e., PLZ3) suspension frequently led to clogging and fiber breakage during electrospinning, affecting the fabrication process (Figure S1A). Fibers’ inconsistent and deformed morphology was obtained. So, we optimized the 1.0 wt % Zn NPs for the fabrication of fibers. The Zn NPs in the fibers were confirmed by SEM-EDS results, illustrating the uniform distribution of particles (Figure 1D). Importantly, the TEM images of the PLZ2 fibers in Figure 1E indicate that the Zn NPs are embedded and distributed well within the fibers.

#### 3.1.2 Controlled Release of Zn^2+^

The release of Zn^2+^ from the fibrous scaffold was confirmed and is depicted in Figure 1F. A higher amount (0.91 μg mL^–1^) of Zn^2+^ was released in PLZ3 at day 2, showing an inconsistent release of the ions up to 14 days. This occurred not only due to the higher amount of Zn NPs but also due to the uneven distribution of particles attached to the surface of the fibers. The controlled release can be observed from PLZ1 (∼0.31 μg mL^–1^) and PLZ2 (∼0.41 μg mL^–1^) on day 2, followed by a downward linear trend up to day 8, after which the release rate became relatively steady by day 14, avoiding burst toxicity while supporting vascularized tissue formation. The amount of Zn^2+^ released was calculated from the total sum of Zn^2+^ from the scaffolds and cell supplement media. In contrast, PLZ0 released a measurable amount of Zn^2+^ (0.09 μg mL^–1^). This may be attributed to the intrinsic Zn content of HDFn cells and the supplemental Zn^2+^ present in the cell culture media.^27^ The optical micrographs in Figure S1D show the result of a colorimetric assay, indicating binding of Zn^2+^ to a ligand, which develops a detectable color shift. A remarkable color change was observed, shifting from yellowish to pink after the addition of the Zn quantifying reagent in the collected media obtained from PLZ0, PLZ1, and PLZ2, indicating a progressive increase of Zn in wound healing, which acts as a therapeutic agent to regulate cellular functions, including immune response and cell proliferation.^28^

#### 3.1.3 Physicochemical Analysis

The XRD analysis verified the incorporation of Zn NPs in the scaffolds, showing characteristic Zn crystalline peaks with a hexagonal shape in PLZ1 and PLZ2, while pure PLGA retained low crystallinity as evidenced by the appearance of a broad crystalline peak (Figure 2A). Further, no significant changes were observed in the chemical structure of PLGA after the addition of Zn NPs, indicating minimal chemical interaction between the metal particles and PLGA, as evidenced by the unchanged FTIR peaks (Figure 2B). In addition, the thermal decomposition of the different scaffolds (PLZ0, PLZ1, and PLZ2) in Figure 2C shows the variation in apparent weight loss curves of the scaffolds at the lower thermal degradation temperature determined by TGA. However, the presence of Zn NPs further reduced the crystallinity of PLGA, as evidenced by the decreased melting point observed in the DSC analysis (Figure 2D). Details are presented in the Supporting Information. Our mechanical testing experiment confirmed that the scaffold’s tensile strength and stiffness increase with Zn content, indicating uniform particle incorporation. The Zn NPs acted as fillers, enhancing interfacial shear strength and stiffness while also potentially improving fiber alignments in electrospinning (Figure S2). Fibers also showed an increase in hydrophilicity once the Zn NPs were incorporated into the fibers, as shown by the lower water contact angle (Figure S3). The particles exposed on the surface of the fiber, as evidenced in the TEM image (Figure 1) that is susceptible to form hydrogen bonding with these particles. Such property in the scaffolds supports cell and fiber interactions at the interface and might enhance cell growth and cell attachments. In addition, these fibrous scaffolds underwent significant degradation in our short-term enzymatic biodegradation experiment (Figure S4). After 1 week of enzymatic exposure, notable changes in fiber morphology, including a fractured fiber texture, were observed, which can be attributed to the enzymatic hydrolysis of PLGA. These properties of the scaffold facilitate the controlled and quantitative release of Zn^2+^. The gradual release of Zn^2+^ from fibrous scaffolds is beneficial for stimulating cell growth, angiogenesis, and antibacterial effects without causing cytotoxicity from a rapid burst of bare Zn.

**Figure 2:**
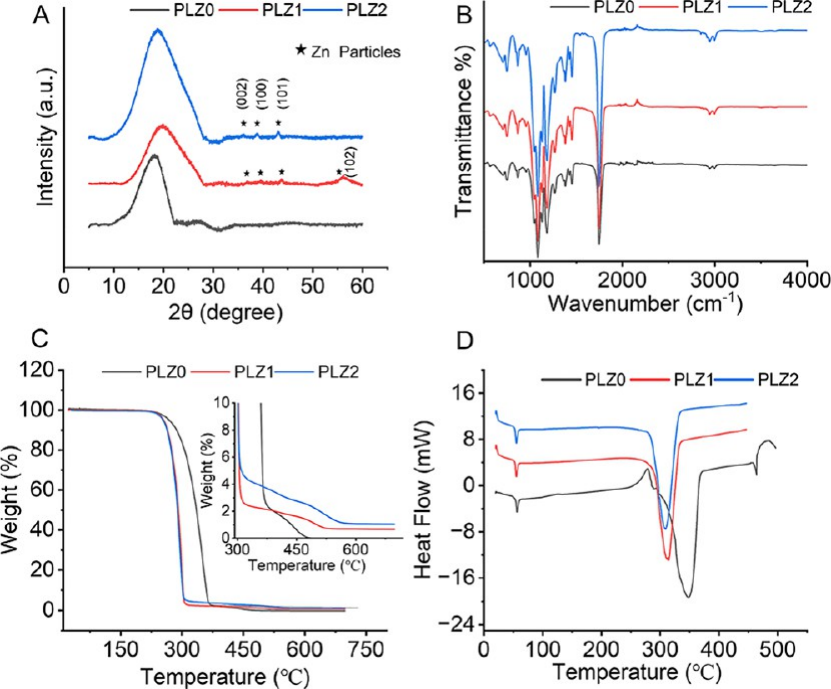
Physicochemical characterization of the fibrous scaffolds. (A) Typical XRD pattern, (B) FTIR spectra, (C) TGA curves, and (D) DSC thermograms.

### 3.2 In Vitro Biocompatibility and Cell Proliferation Study

The effect of the different compositions of Zn-electrospun scaffolds (PLZ1, PLZ2, and PLZ3) was examined on HDFn and HUVEC, as shown in Figure 3A,C with MTT assay and AB assay, Figure 3B,D. The HDFn and HUVECs grown on scaffolds showed no significant increment in cell proliferation and growth at day 1 among PLZ0, PLZ1, and PLZ2, while in PLZ3, the cell compatibility dropped remarkably due to a higher amount of Zn^2+^ released from the scaffold in the cell culture medium. As the incubation days reached 5, cell proliferation increased dramatically on the PLZ2 scaffold compared to PLZ0 (*p* < 0.001). The Zn-based scaffold has the potential to enhance cell growth and survival by raising extracellular protein expression, given that Zn is a predominant element in various human tissues and plays a critical role in numerous biological processes. To create the new tissue required to heal a wound, Zn is a cofactor for enzymes involved in collagen and protein synthesis, growth, and movement of cells like fibroblasts and keratinocytes.^29^ The matrix metalloproteinase MMP-9 is involved in wound healing, especially during the early phases of tissue degradation and inflammation. While high MMP-9 activity can hinder wound closure and lead to chronic wounds, it is necessary for debris removal and cell migration.^30^ Accordingly, PLZ3 scaffold downregulates cell proliferation and differentiation in our study. The results obtained from the MTT assay suggest that the proliferation rate on PLZ2 was statistically significant (*p* < 0.001 and *p* < 0.01) on day 5, compared with PLZ0 and PLZ1 scaffolds, respectively. The enzymatic roles of Zn^2+^ enhance the metabolic activities of cells, confirming the higher number of cell survivals and proliferations.^20^

**Figure 3:**
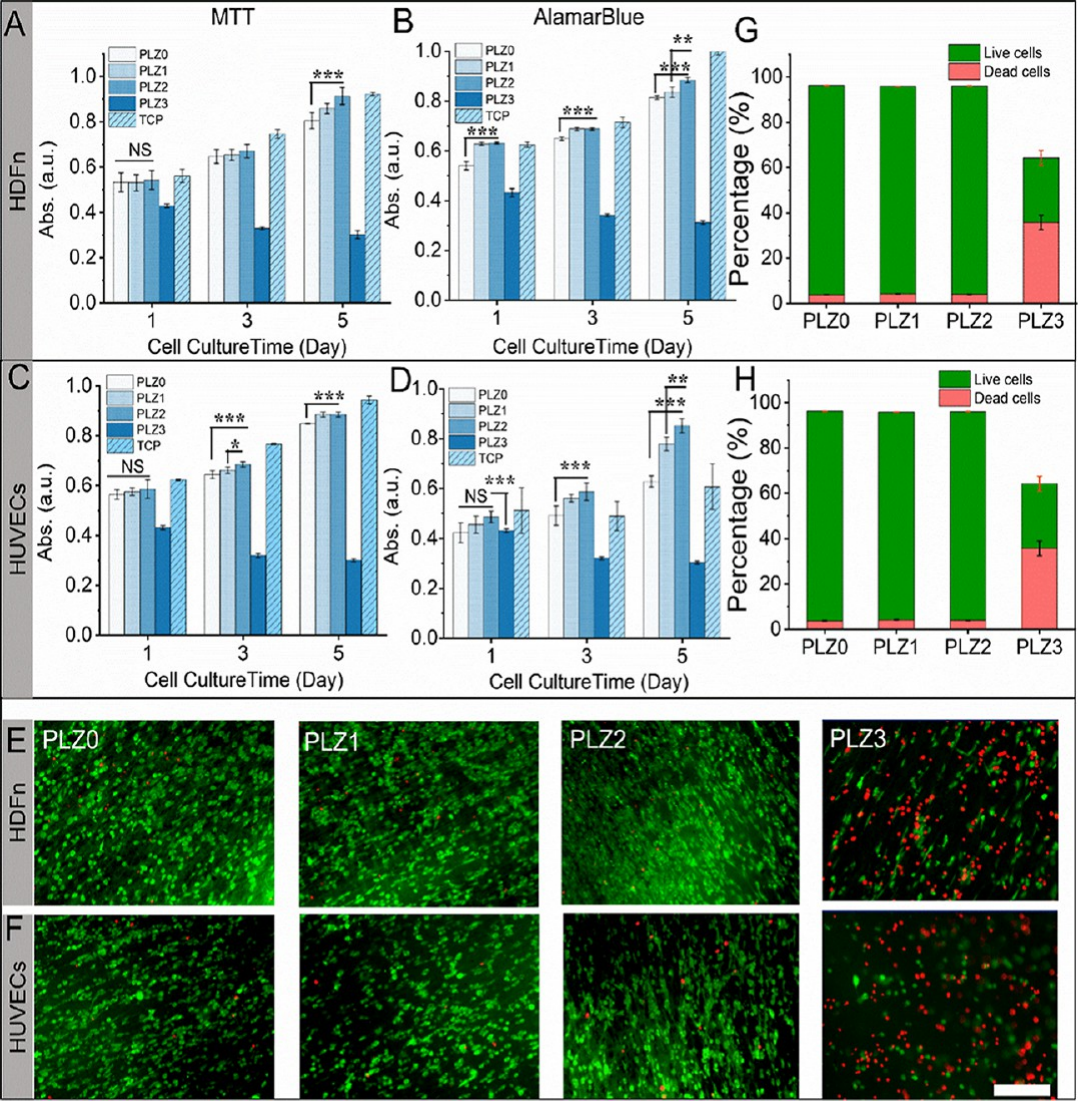
In vitro performance of fibrous scaffolds for HDFn and HUVEC survivability and proliferation. (A and C) Evaluation of the metabolic activity of HDFn and HUVECs via MTT assay. (B and D) Evaluation of cell viability via AB assay of the different scaffolds at days 1, 3, and 5. Fluorescence microscopy images of cells stained with live/dead staining dye at day 5 of the culture (E and F). Live cells (green) and dead cells (red) stained using PerkinElmer LLC via Acridine Orange/Propidium Iodide (AOPI) staining solution. Scale bar = 100 μm. (G and H) The percentage of live and dead cells obtained from respective images (E and F) using the ImageJ software. One-way ANOVA with Tukey’s post hoc test was used to perform statistical significance, and data were expressed as the mean ± SD; *n* = 4 per group for the cell viability test (where NS = no significance, *p < 0.05, **p < 0.01, and ***p < 0.001).

The AB assay showed increased cell proliferation over time, consistent with MTT results. There was an increase in the metabolic activity of cells in the Zn-based scaffolds (PLZ0 < PLZ1< PLZ2) as the incubation days increased, a similar trend to the control (tissue culture plate-TCP). However, in the PLZ3 scaffold, the metabolism decreased significantly. Indeed, zinc, as a key cofactor, supports fibroblast growth, proliferation, and migration in vitro. The proliferation of HDFn via in vitro models becomes a powerful clue in wound healing and tissue regeneration that can complement animal-based research. The continuous proliferation and growth of HDFn on PLZ1 and PLZ2 indicate ECM formation and tissue remodeling. Further, PLGA scaffolds embedded with Zn degrade continuously confirmed by the controlled release of Zn^2+^, allowing for the extension of the Zn dose up to 1 wt % to promote HDFn and HUVEC regeneration. In the meantime, it has been reported to have cytotoxicity to BMSCs, despite its controlled release of not more than 2.055 ± 0.223 μg mL^–1^ of Zn at 16 weeks from the PLGA/β-TCP/Zn scaffold.^31^ Notably, the proliferation and regeneration of HDFn cells on the PLZ1 and PLZ2 scaffolds retain an inherent capacity to become a suitable platform for cell engraftment and wound-healing applications. The controlled release of Zn^2+^ from the scaffold may regulate Zn uptake by cells, allowing anti-inflammatory and immune-responsive properties. The live/dead fluorescence images in Figures 3E,F and S5 indicate that the scaffolds with up to 1.0 wt % of Zn NPs have good biocompatibility and bioactivity to HDFn and HUVECs. The extensive, dense green fluorescence images suggested a higher mass of alive and healthy cells exhibiting cytocompatibility. The results are in agreement with the results of MTT and AB assays, where the absorbance was proportional to the number of viable cells. Notably, the cell density in all fibrous scaffolds was significantly increased (Figure 3G,H). More than 95% of cells survived on scaffolds. In contrast, the 2.0 wt % Zn scaffold showed cytotoxicity to both cells. The concentration of Zn^2+^ released from the scaffolds varies over time among the groups at 37 °C and normal pressure of about 744 mm Hg, which was confirmed from a colorimetric assay. The concentration of the ions released from the scaffolds with 1 wt % of Zn was determined, showing a positive effect on cellular growth and proliferation, and was considered the optimal amount of Zn in scaffolds.

### 3.3 Cell Attachment and Morphology of HDFn and HUVECs on Fibrous Scaffolds

The fluorescence microscopy images in Figures 4 and S6 illustrate the influence of Zn^2+^ on cell adhesion and proliferation at days 1 and 3. On day 1, both HDFn and HUVECs were observed to be flat and slightly elongated on the PLZ0 and PLZ1, while cells on the PLZ2 were observed to be elongated and extended along with fiber alignment, suggesting that the scaffolds showed a strong integrity of biointerface for cell attachment. The cells retained their shape to resemble the actual morphology of HDFn and HUVECs in vitro under physiological conditions on day 3. The cellular mass with directional migration exhibited interconnected, narrow dendrite-like projections over time. The normal and healthy adherent cells were extensively spread throughout the scaffold surface, with extensive outgrowth of filopodia. The healthy cells indicate strong bioactivity with an appropriate surface roughness and microenvironment of the scaffolds that support cellular functions, such as cell attachment, development, cell-to-cell interaction, and communication. Undoubtedly, the controlled release of Zn^2+^ as a micronutrient plays a biocatalytic role in regulating protein expression, activating cellular metabolism, enhancing immune functions, cell signaling, and cell division.^32^ The quantification of the fluorescence intensity of the cytoskeleton and nuclei in Figure 4A,B varies on different scaffolds. An increased expression of proteins in the cytoskeleton was observed in the order of PLZ0 < PLZ1 < PLZ2 on day 1 and day 3 in both cell types, indicating well growth with a healthy cell population. Following 3 days of cultivation, the intensity of the proteins in nuclei was also significantly increased in PLZ2 compared to PLZ0 (*P* < 0.05), Figure 4A. Additionally, in Figure 4B, the intensity of protein in nuclei and cytoskeleton was significantly increased in PLZ2 compared to PLZ0 (*P* < 0.01 and *P* < 0.05, respectively). The release of Zn^2+^ from the scaffolds could maintain intracellular and extracellular Zn^2+^ homeostasis through Zn transporters for cellular activities and biological responses.^33^ Notably, the Zn^2+^ with a range from 1 μM to 50 μM enhanced proliferation and osteoblastic activity, while for the immune cells, a high concentration of Zn levels, ranging from 10 to 29 mM in insulin, encourages cells to proliferate, ECM deposition, and immune response.^34^ The Zn wt % and the ions released from PLZ2 scaffolds suggest that our materials could be the source of microminerals to HDFn and HUVECs’ survivability, migration, and differentiation. Indeed, cell organization on the fibrous scaffolds showed remarkably different patterns compared to the control, TCP (Figure S6). The cell structures and outgrowth of actin filaments exhibited a flattened and polygonal shape with a wide cellular membrane, while an elongated, well-established, and spindle-shaped cellular structure with cell–cell connections was observed on the fibrous scaffolds. This confirms that the suitable topography of the scaffolds supports cell growth and development.

**Figure 4:**
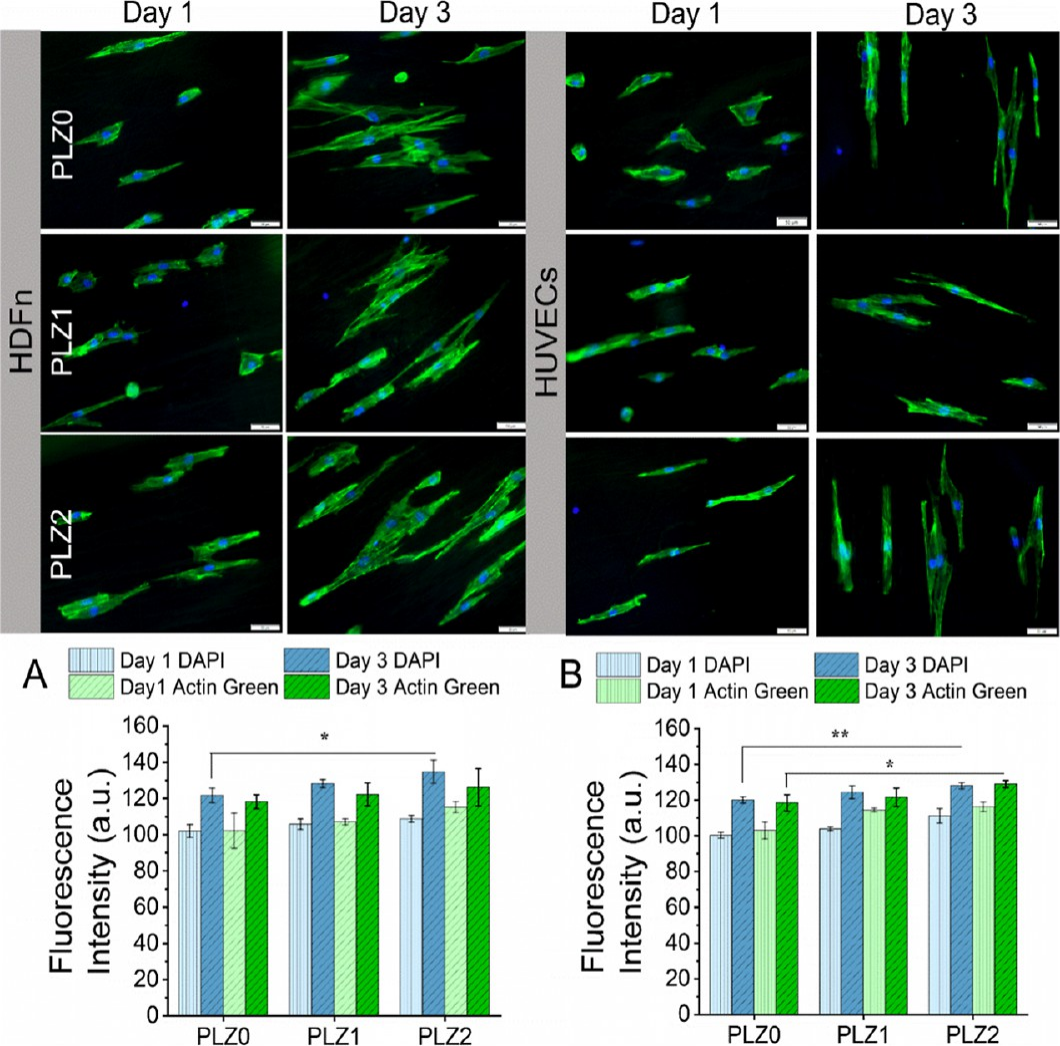
Fluorescence microscopy images of cells cultured on the different fibrous scaffolds. Morphology after day 1 and day 3. Left: HDFn cells and right: HUVECs. Cytoskeleton stained with Actin green 488 (green) and nuclei counterstained with DAPI (blue). Scale bar = 50 μm. (A and B) The fluorescence intensity of the cytoskeleton and nuclei of the respective images (*n* = 3), HDFn, and HUVECs, was analyzed using ImageJ software. One-way ANOVA with Tukey’s post hoc test was used to calculate statistical significance, and data were expressed as the mean ± SD; *n* = 3 per group (where *p < 0.05 and **p < 0.01).

### 3.4 Differentiation Study of HDFn through Immunocytochemistry Analysis

The immunofluorescence microscopy images in Figures 5, S7, and S8 demonstrate the expression of the cytoplasmic proteins vimentin, an intracellular structural protein, and α-SMA, a marker of activated myofibroblasts. The expression of these proteins confirms the differentiated HDFn and its changes to myoblasts. Particularly, the expression of vimentin notifies the growth and development of the mature cell type along with its differentiation phase. The differentiated HDFn expressed the intracellular network of filamentous protein on different scaffolds. The expression of proteins was significantly higher on PLZ2 when compared to PLZ0 on 7 and 14 days. Primarily, these proteins are composed of a strong filament-like network that spreads from the nuclear periphery to the membrane and protects cell nuclei from external injury.^35^ The cells’ protection against distortion in the structural organization of the cytoplasm is ensured by the assembly of widespread filaments, vimentin. This protein was organized in an extended fibrillar assembly ensheathed with actin filaments, indicating that the cells undergo a differentiation and physiological alternation process. The scaffolds provide a microenvironment that offers structural and functional support for the cells to mediate the expression of vimentin to the cell membrane while spreading. Moreover, this protein binds to phosphorylated ERK and RhoK, modulating MAPK cascades and actin organization, which helps in cell migration, attachment, and cell signaling.^36^ The scaffolds with Zn NPs accelerated the expression of vimentin for the regulation of TGF-β1 and slug signaling that stimulates HDFn for ECM synthesis and cell-to-cell communications for facilitating wound healing and tissue regeneration.^37^ The fluorescence in intermediate filaments is induced by the existence of extracellular Zn that can interact with vimentin-cystine to form a metalloprotein complex. Predominantly, immune, endothelial/epithelial, and undifferentiated muscle cell growth and repair of wounds are stimulated by the combination of Zn and vimentin. Thus, a higher expression of vimentin was observed in immunofluorescence images of HDFn cells, suggesting that PLZ2 is effective for cell differentiation and regeneration.

**Figure 5:**
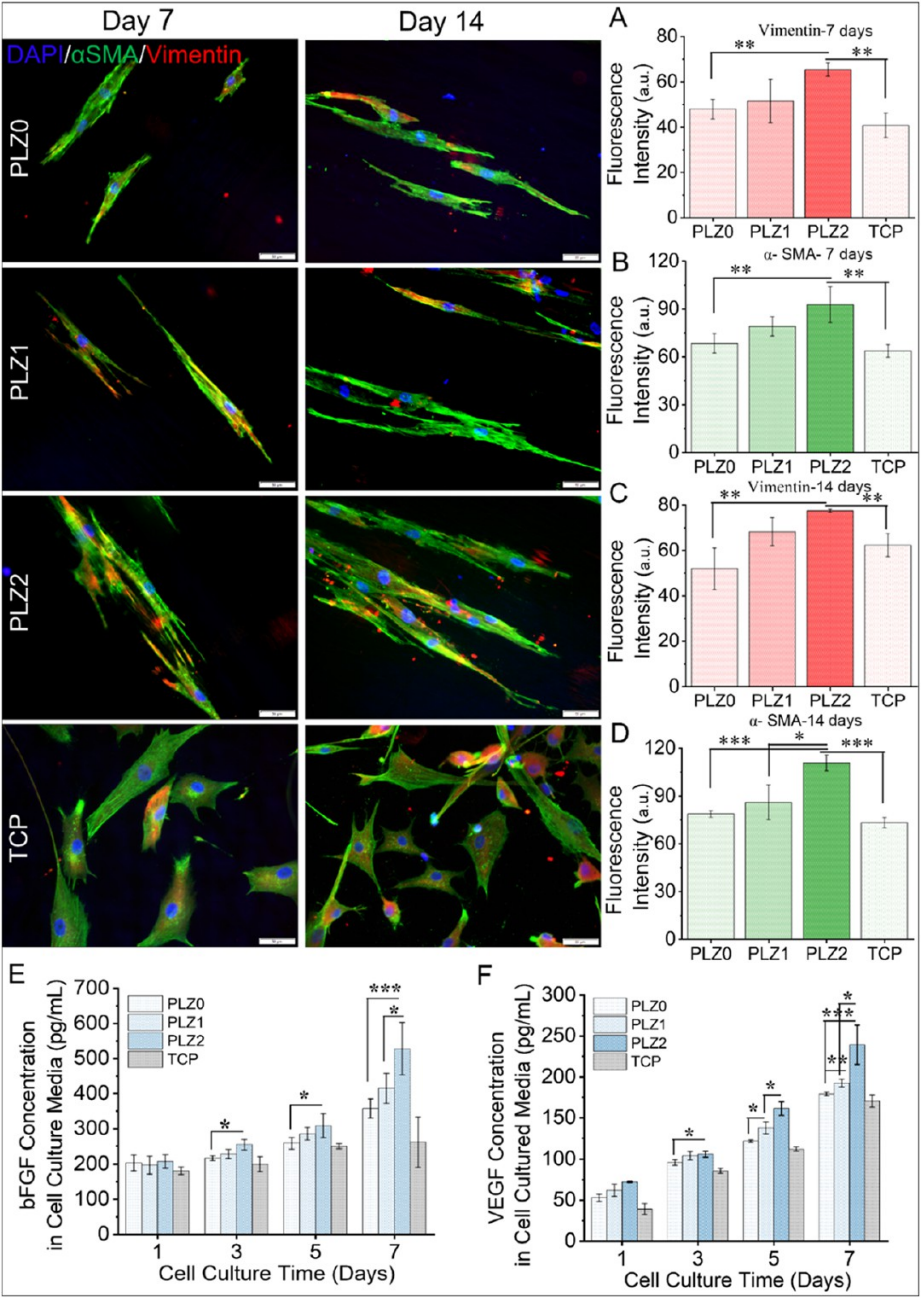
Expression of different proteins after transformation of the fibroblasts into myofibroblasts and differentiated HDFn cells. Left and middle panel: immunofluorescence images of intracellular vimentin (red) and α-smooth muscle actin (α-SMA) (green) expressed by HDFn cells after incubation for 7 and 14 days on different fibrous scaffolds labeled using immunocytochemistry. Scale bar = 50 μm. (A and B) and (C and D); the fluorescence intensity of the protein markers, vimentin, and α-SMA for days 7 and 14, respectively, was analyzed using ImageJ software. Quantification of growth factor (E) bFGF and (F) VEGF concentration in cell-cultured media on predetermined days (1, 3, 5, and 7). One-way ANOVA with Tukey’s post hoc test was used to calculate statistical significance, and data were expressed as the mean ± SD; *n* = 4 per group for the cell viability test (where *p < 0.05, **p < 0.01, and ***p < 0.001).

Additionally, the expression of α-SMA indicates the transformation or modulation of HDFn into myofibroblasts (Figure 5). The expression of α-SMA by HDFn confirms the inherent biochemical properties of the scaffolds. The fluorescence images showed a higher expression of α-SMA on day 14 than on day 7. The strong expression of α-SMA crossing the cytoplasm within the vimentin indicates the cell transformation and cell trans-differentiation from fibroblast into activated myofibroblasts. The expression of α-SMA specifies the differentiation of NIH3T3 into myofibroblasts, which undergo contractility and apoptosis to lead the wound healing process.^38^ The Zn^2+^ stimulates the fibroblast to accelerate wound healing ability by upregulating cell-cycle proteins, p38-MAPK, and ERK1/2 signaling pathways, reaching their maximum metabolic rate in 2 to 4 days.^39^ In addition, the amount of Zn higher than 100 μM inhibits the expression of α-SMA and suppresses the TGF-β signaling pathway significantly.^40^ The PLZ2 scaffolds that gradually degraded with time and released a controlled dose of Zn^2+^ enhanced the expression of α-SMA. This controlled release of Zn^2+^ prevents its excessive accumulation, regulates Zn uptake, and supports anti-inflammatory and immune responses. The higher expression of α-SMA by HDFn confirms the efficacy of PLZ2 for promoting long-term tissue healing and remodeling.

Furthermore, the level of vimentin and α-SMA was quantified from the corresponding immunofluorescence images, as shown in Figure 5A–D. The immunofluorescence intensity of vimentin varies on different scaffolds. A trend of increased expression was observed (PLZ0 < PLZ1 < PLZ2) on day 7 and day 14, indicating that the cells were highly proliferated and either activated or modulated into myofibroblasts. In contrast, less expression was observed in the control (TCP). Notably, the expression of vimentin significantly increased (*p* < 0.01) on PLZ2 compared to PLZ0 and TCP. This heightened expression also indicates the epithelial-mesenchymal changes, which relate the normal fibroblast differentiation to activate fibroblast cells in association with mechanical variation and filamentous stiffness.^41^ The expression of the cytoskeleton proteins stimulates cell motility, maintains cell shape, and ensures the integrity of the cytoplasm in the cytoskeleton.^42^ The higher expressions of α-SMA and vimentin on PLZ2 scaffolds may be attributed to the presence of Zn, which regulates cellular and physiological processes. These proteins are proportionally correlated and regulate the ability of fibroblasts to transform into differentiated myofibroblasts. The release of Zn^2+^ from the scaffolds could follow the transfer path from the extracellular environment to the cytoplasm, thereby preventing cell apoptosis and promoting cell metabolic activities. Zn supplementation is a particularly crucial trace micronutrient for cells, and Zn^2+^ plays a pivotal role in various cellular processes as a component of transcription factors and epigenetic modulators, cell differentiation and could also advance comprehension of the injured cells/tissue healing mechanism.^43^ Hence, the results suggest that Zn micronutrients in fibrous scaffolds enhance protein expression in HDFn. This indicates that the scaffolds support wound healing metabolism, which could be advantageous in effective preclinical and biomedical applications.

### 3.5 Secretion of bFGF and VEGF in Cell Culture Medium

The secretion of bFGF and VEGF in the fibroblast culture medium was quantified (Figure 5E,F). VEGF, a pro-angiogenic factor, encourages microvascular hyperpermeability and precedes angiogenesis. The level of bFGF and VEGF secretion by HDFn was stimulated by Zn^2+^. A significant increment of bFGF and VEGF was observed, which was secreted by HDFn cultured in the Zn-based scaffolds on day 7. Overall, the expression level of bFGF and VEGF in PLZ2 was statistically significant (*P* < 0.05) at days 3, 5, and (*P* < 0.001) at day 7 compared to PLZ0. The expression of bFGF (1.47-fold) and VEGF (1.34-fold) was higher in PLZ2 than in PLZ0 on 7 days. The increased level of growth factors suggests that the positive activity of Zn^2+^ induces cell growth, proliferation, and differentiation of the HDFn. Zn homeostasis regulates DNA synthesis, hormonal growth, cell division, and proliferation in association with VEGF-mediated signal transduction and secretion of cell growth factors.^44^ The increase in the bFGF and VEGF levels in the conditioned medium was observed not only because of HDFn but also due to the stimulatory effects of Zn^2+^ present in the scaffolds that induced HDFn to stimulate growth factors.

### 3.6 In Vitro Macrophage Cell Proliferation and Macrophage Polarization

To explore macrophage proliferation and its functional phenotypes, a representative diagram (Figure 6A) was depicted, which was confirmed through MTT and AB assays (Figure 6B,C). Similar and increased trend of cell proliferation rate over time (1, 3, and 5 days). On day 1, cells proliferated uniformly without a significant difference being observed on PLZ0, PLZ1, and PLZ2, but on days 3 and 5, the proliferation of cells and their metabolic activities increased significantly compared to day 1. The PLZ1 and PLZ2 showed the potential to enhance the growth and survival of immune cells. Notably, Zn stimulates the expression of pro- and anti-inflammatory proteins and cytokines to induce cellular proliferation and differentiation, which also has modulatory functions to transform M1 to M2 macrophages.^45^

**Figure 6:**
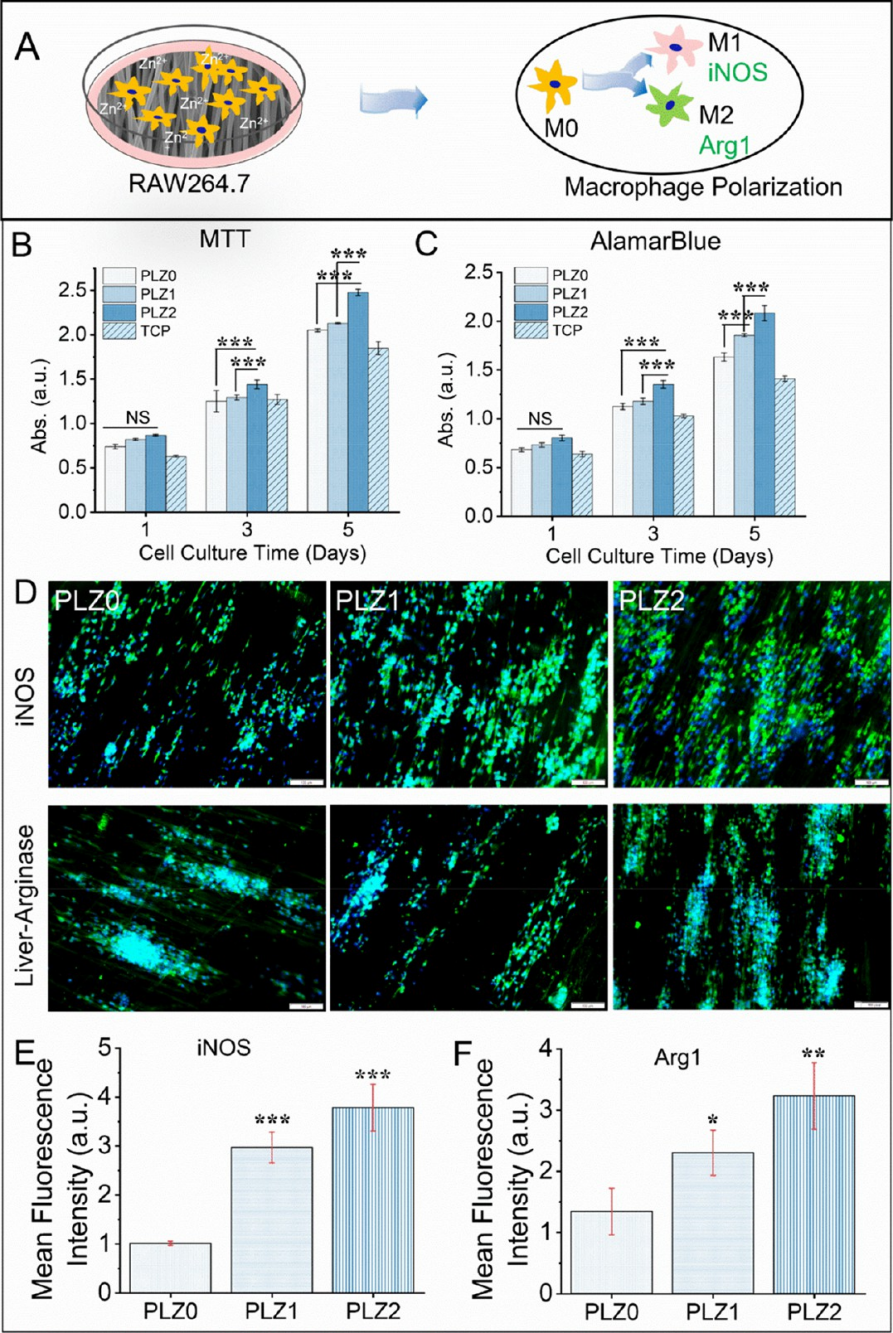
In vitro performance and immunomodulatory activity of different fibrous scaffolds with RAW264.7 cells cultured at different time intervals. (A) Schematic illustration depicting the polarizing effect of Raw264.7 cells cultured on the scaffolds. (B) Evaluation of the metabolic activity via MTT assay. (C) AlamarBlue activity to determine cellular viability of the different scaffolds at days 1, 3, and 5. (D) Immunofluorescence images of macrophage polarization markers–iNOS and Agr1. Scale bar = 100 μm. (E and F) The intensity of the markers of respective immunofluorescence images was analyzed using the ImageJ software. One-way ANOVA with Tukey’s post hoc test was used to calculate statistical significance (mean ± SD; *n* = 4 per group for cell viability test, where NS = no significance, *p < 0.05, **p < 0.01, and ***p < 0.001).

Further, the polarity of macrophages was confirmed by immunofluorescence staining of macrophage polarization markers–iNOS and Agr1 (Figures 6D, S9, and S10). The macrophages on the scaffolds (PLZ1 and PLZ2) exhibited a higher expression of iNOS. However, iNOS expression is weak on the PLZ0 scaffold. Meanwhile, the expression of Arg1 (expressed by M2 macrophages) is also noted to be higher in the PLZ1 and PLZ2 scaffolds. The potential of Zn-based scaffolds to regulate macrophage polarization for tissue engineering has been explored, and it has been found that Zn-induced macrophage polarization—M1 macrophage, the pro-inflammatory phenotype, and M2 macrophage—is less inflammatory and promotes tissue repair.^46^ M1 polarization of the macrophage was observed due to an acute stress response to a sudden release of Zn^2+^. The studies have shown that Zn^2+^ stimulated the up-regulation of phenotypic markers like iNOS expression in macrophages, but the appropriate Zn^2+^ concentrations cause macrophage polarization from M1 to M2.^47,48^ Moreover, the level of macrophage polarization protein markers–iNOS and Agr1 was quantified by analyzing the corresponding immunofluorescence images (Figure 6E,F). As illustrated, the immunofluorescence intensity of protein expression varies. An ascending order of expression was observed (PLZ0 < PLZ1 < PLZ2), indicating that cells were highly activated and polarized. Notably, the iNOS expression is significantly increased (*p* < 0.001) on PLZ1 and PLZ2 compared to PLZ0. Similarly, expression of Arg1 was also increased significantly (*p* < 0.05 and *p* < 0.01). The findings confirm the transformation of macrophages into M1 macrophages followed by the M2 phenotype.

### 3.7 Indirect Coculture of HUVECs with HDFn

HUVECs grown on a CCM obtained from HDFn cultured on different scaffolds showed excellent cell growth and proliferation. The schematic diagram in Figure 7A illustrates the indirect coculture method for the evaluation of the MTT assay and AlamarBlue (Figure 7B,C). Proliferation of HUVECs on PLZ0, PLZ1, and PLZ2 was significantly higher on days 3 and 5 compared to day 1. The synergistic effect of biocompatible PLGA, Zn NPs, and the growth factors released from HDFn enhanced cell viability. Importantly, Zn^2+^ and the cell growth factors stimulated the HUVECs for proliferation and enhanced cellular activities. The cell density in all scaffolds was higher with an increasing incubation period. However, MTT and AB assay data were similar in both Zn-based CCM on day 5 compared to the monoculture study (above-mentioned in Figure 3C,D). At day 5, the proliferation of cells showed a significant difference on PLZ0 and PLZ1 (*p* < 0.001), PLZ0 and PLZ2 (*p* < 0.001), and PLZ1 and PLZ2 (*p* < 0.01) obtained by AB assay results. This might be due to the growth factors and ECM components, such as matricellular proteins and dermatopontin fibril-associated collagens with interrupted triple helices (FACIT collagens) produced by HDFn.^49^ Further, the effect of the CCM on HUVECs showed no cytotoxicity at different incubation periods. The CCM showed a positive effect in improving the metabolic activity of HUVECs along with the growth factors (bFGF and VEGF) secreted by HDFn.^50,51^ In tissue constructs, the coculture method is highly acceptable, where soluble growth factors produced by one type of cell are capable of influencing the metabolic activity of other cell types.

**Figure 7:**
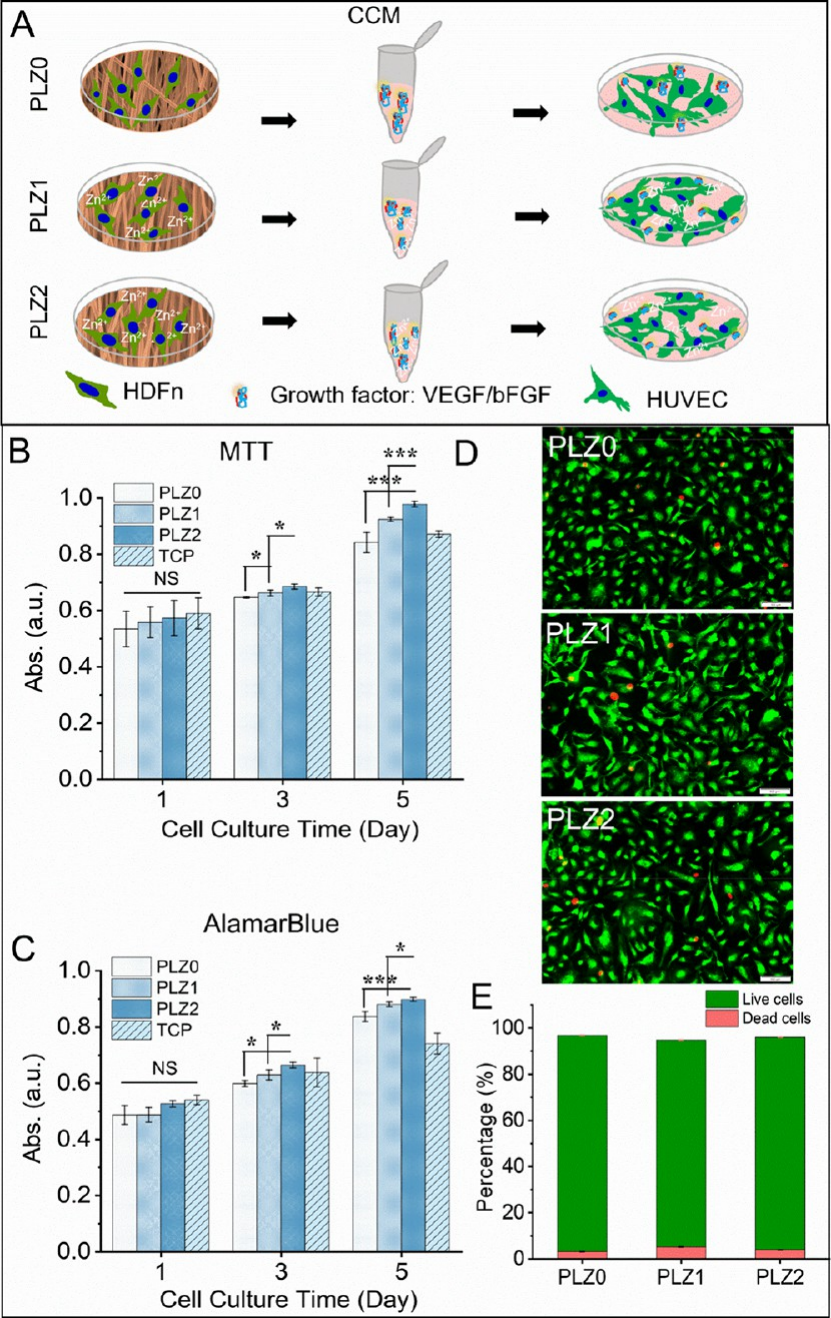
(A) Schematic illustration of indirect coculture. In vitro, cell viability, and cytotoxicity of indirect coculture of HUVECS on conditioned culture media from HDF cultured on different fibrous scaffolds. (B) Evaluation of the metabolic activity of HUVECs on conditioned medium via MTT assay. (C) AlamarBlue activity to determine cellular viability at days 1, 3, and 5. (D) Live cells (green) and dead cells (red) stained using AOPI staining solution. Scale bar = 100 μm. (E) The live and dead cells’ percentage obtained from respective fluorescence images (D) using ImageJ software. Fluorescence microscopy images of cells stained with live/dead staining dye at day 5 of the culture. One-way ANOVA with Tukey’s post hoc test was used for statistical significance, and the data were expressed as the mean ± SD; *n* = 4 per group (where NS = no significance, *p < 0.05, **p < 0.01, and ***p < 0.001).

The live and dead cell staining results revealed the cytocompatibility of CCM (Figure 7D). The fluorescence micrographs showed a lower number of cell deaths in the CCM, which is similar to the dead cells in TCP (Figure S11), suggesting that the microenvironment favors cell growth, proliferation, and spreading. The percentage of live and dead cells was obtained from respective fluorescence images (*n* = 3), as shown in Figure 7E. Cell survivability was observed to be over 97% when incubated with different CCMs, confirming that an indirect coculture microenvironment accelerates cell growth and development.

### 3.8 In Vitro Angiogenesis Study of HUVECs and Their Responses to CCM

#### 3.8.1 Cell Migration/Scratch Assay

To evaluate the angiogenic potential of HUVECs, cell migration and tube formation experiments were performed. Cell migration is an important progression of blood vessel development and vascular restoration.^52^ An in vitro wound-healing assay was examined on a cellular monolayer to evaluate the closure rate of an artificially generated wound in two different models. The optical images in Figure 8A,B illustrate the scratch-wound healing process over 24 h. The proliferation and migration of HUVECs cultured on the extracts and the conditioned medium were improved compared to freshly prepared medium (FM) and CCM obtained from TCP (CM). The migration rates of HUVEC increased significantly in the extracts from Zn-based scaffolds compared to PLZ0 and TCP. Zn regulates endothelial cells’ mobility, modulates cellular signal recognition, and stimulates cells to spread, proliferate, and migrate.^53^ It helps endothelial tubule formation and cytoskeletal reorganization. The cells’ actin cytoskeleton organization and cell shape determine the effect of Zn^2+^ on cellular processes.^54^ The HUVECs cultured with CM demonstrated much denser cell proliferation and migrated toward the scratched area (Figure 8B). Figure 8C shows the remaining percentage of wound closure after 24 h of incubation of cells with the extract of PLZ0, PLZ1, PLZ2, and FM, corresponding to 29.45%, 25.56%, 24.06%, and 31.02%, respectively. In addition, cell-free areas of 24.26%, 20.94%, 14.12%, and 27.19% were measured corresponding to the CCM of PLZ0, PLZ1, PLZ2, and CM, respectively (Figure 8D). These results support that Zn-stimulated wound healing with angiogenic activation occurs in defect areas. The Zn^2+^ enhanced cell viability and proliferation, which increased the level of VEGFA. VEGFA precisely stimulates endothelial cells and has numerous effects, together with facilitating improved vascular permeability. It encourages angiogenesis, vasculogenesis, and endothelial cell growth, promotes cell migration, and inhibits apoptosis.^55^ The increase in the percentage of cell progress in CCM indicates that the growth factors secreted by HDFn are responsible for the growth, proliferation, and migration of HUVECs.^56^ Noticeably, significant promotion in cell migration was observed on all Zn-containing scaffolds, showing dependence on the amount of Zn^2+^. We found that ∼75% scratched area was covered by migrated cells in the Zn-based extracted groups, while ∼86% was observed in CCM containing Zn^2+^. These results suggest that the recovery rate of the HUVECs also depends on the concentration of Zn^2+^ and becomes a therapeutic agent for the treatment of injured tissue.

**Figure 8:**
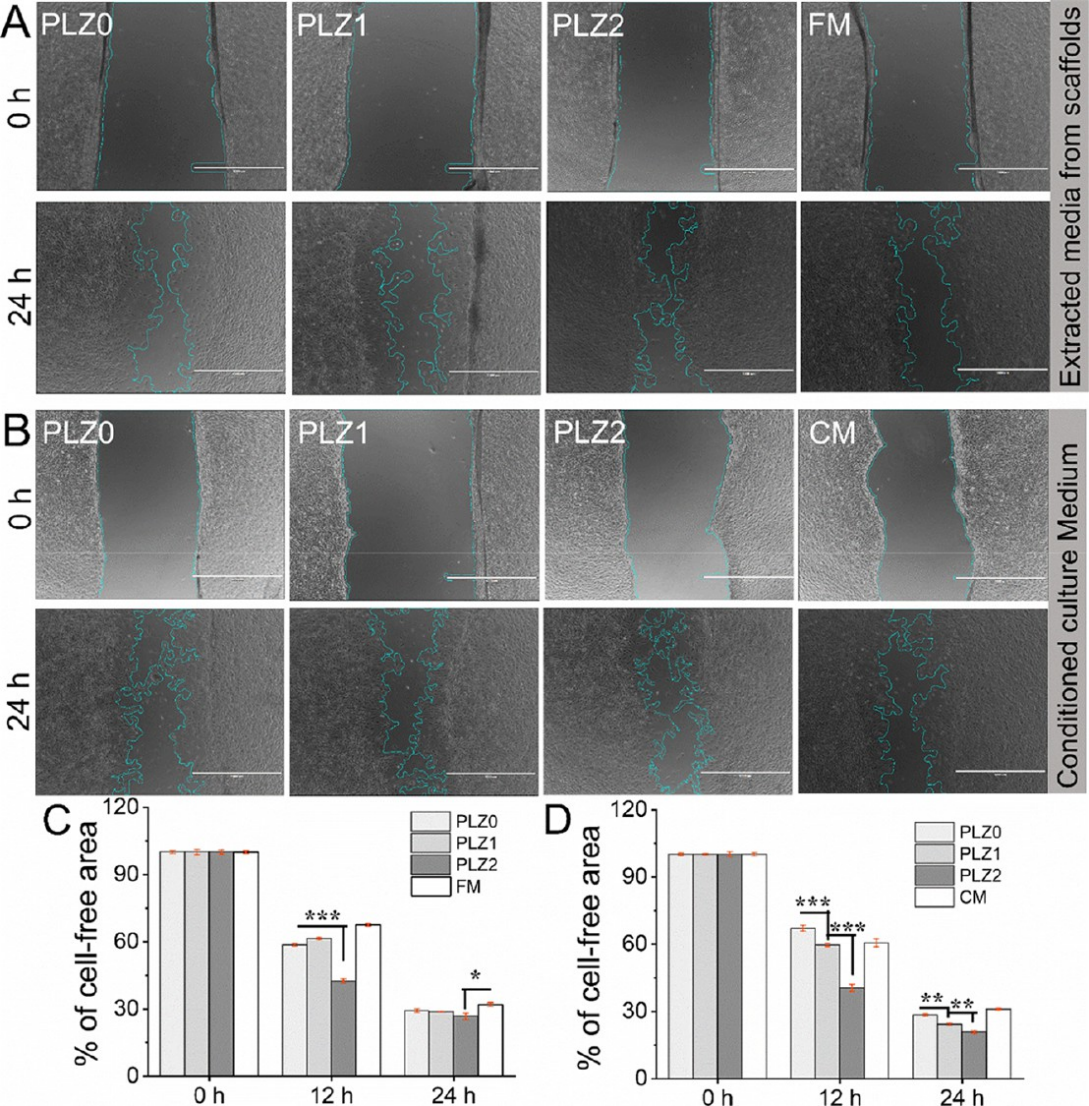
In vitro scratch-wound healing/migration assay. (A,B) Microscopy images of the cells’ migration toward the wound area, observed for the HUVECs cultured on extracted media from different fibrous scaffolds, and HUVECs cocultured on the conditioned culture media from the HDFn cultured on different scaffolds, respectively. A phase contrast inverted microscope was used to obtain images at 0 and 24 h. The migratory capability of HUVECs was measured using a wound healing assay tool with the ImageJ software. Scale bar = 1000 μm. (C,D) The bar graph illustrates the percentage of cell-free area at the indicated time during the scratch wound assay. Data were analyzed using the one-way ANOVA with Tukey’s post hoc test and expressed as the mean ± SD; *n* = 3 per group for statistical significance (where *p < 0.05, **p < 0.01, and ***p < 0.001).

#### 3.8.2 Tube Formation/Neovascularization

The microscopy images in Figures 9A and 10A show the angiogenic potential of HUVECs on extracts of different scaffolds and different CCMs. After HUVECs were seeded onto the matrigel matrix with extract and CCM, tube-like aggregation was noted as early as 3 h. These images showed a profound formation of vessel-like networks and branches at early time points (3–6) h on extracts and CCM. The formation of new blood vessels for remodeling tissues and organs is important during angiogenesis in early wound healing, which involves the supply of necessary nutrients and oxygen to the fibroblasts and regulates their activity.^1^ Meanwhile, the fibroblasts’ survivability, proliferation, and differentiation promote the formation of new connective tissue or granulation tissue with wound-healing processes.^57^ The endothelial tubular network formation encompasses the tasks involved in angiogenesis. The endothelial cells seeded on a layer of basement membrane matrigel matrix quickly transformed and reorganized to form tubular structures, cellular junctions, mesh networks, nodes, and branches across the matrix. This tube formation assay provides an additional qualitative and quantitative evaluation of the cell behavior. The angiogenesis ability of HUVECs was quantitatively measured in extracts of different scaffolds and in CCM, Figures 9B–G and 10B–G, respectively. The extract of the PLZ1 and PLZ2 induced HUVECs’ ability to form tubes. The length of the tubes was measured as 10847.7 ± 262.7 pixels for PLZ1 and 12639.3 ± 1403.1 pixels for PLZ2, which are significantly greater than PLZ0 (*p* < 0.05 and *p* < 0.001) and FM (*p* < 0.001). Further, the PLZ2 extract showed a significant increment (*p* < 0.05) of node numbers (339.6 ± 28.9), (*p* < 0.01) junctions (97.6 ± 8.9), (*p* < 0.05) segment length (8799 ± 396.5, pixels), and (*p* < 0.001) branching length (12043.3 ± 288.6 pixels) compared to PLZ0. The network of cell–cell junctions and tube alignment confirms that the endothelial cells were differentiated into neovascularization. Considering that the formation of new blood vessels requires mobility, the improved migration capabilities noted with Zn^2+^ may contribute to cellular organization in tube structures. Zn^2+^ regulates endothelial activity, such as new blood vessel formation, vascular cell survival/growth, and intracellular signaling pathways.^54^ A majority of cells were seen in smaller clusters rather than the tube extensions in PLZ0 and FM compared to PLZ1 and PLZ2, which may be because of the lack of cell-to-cell interactions. In CCM samples (Figure 10B–G), the number of meshes, nodes, junctions, tube length, branching length, and master segment length seemed significantly higher (*p* < 0.01 or *p* < 0.05) in PLZ2 compared to PLZ0 and PLZ1. The bFGF and VEGF secreted by HDFn indirectly induced HUVECs’ growth, differentiation, and neovascularization. The family of VEGF has a crucial role in embryonic development in vasculogenesis and angiogenesis.^58^ Cell-to-cell interactions and their consistent communication are important in stimulating the soluble growth factors for self-assembled network formation, which has a potential application for vascularization. However, the specific quantity of VEGF required to stimulate neovascularization and therapeutic angiogenesis is currently unknown. Indeed, the delivery of growth factors is essential for the implementation of therapeutic angiogenesis and the successful development of blood vessels in ischemic tissue. Hence, the coculture method is thereby chosen as a better approach than growth factor delivery for cell growth and even supports the formation of new connective tissues that help in complete wound repair through tissue regeneration.

**Figure 9:**
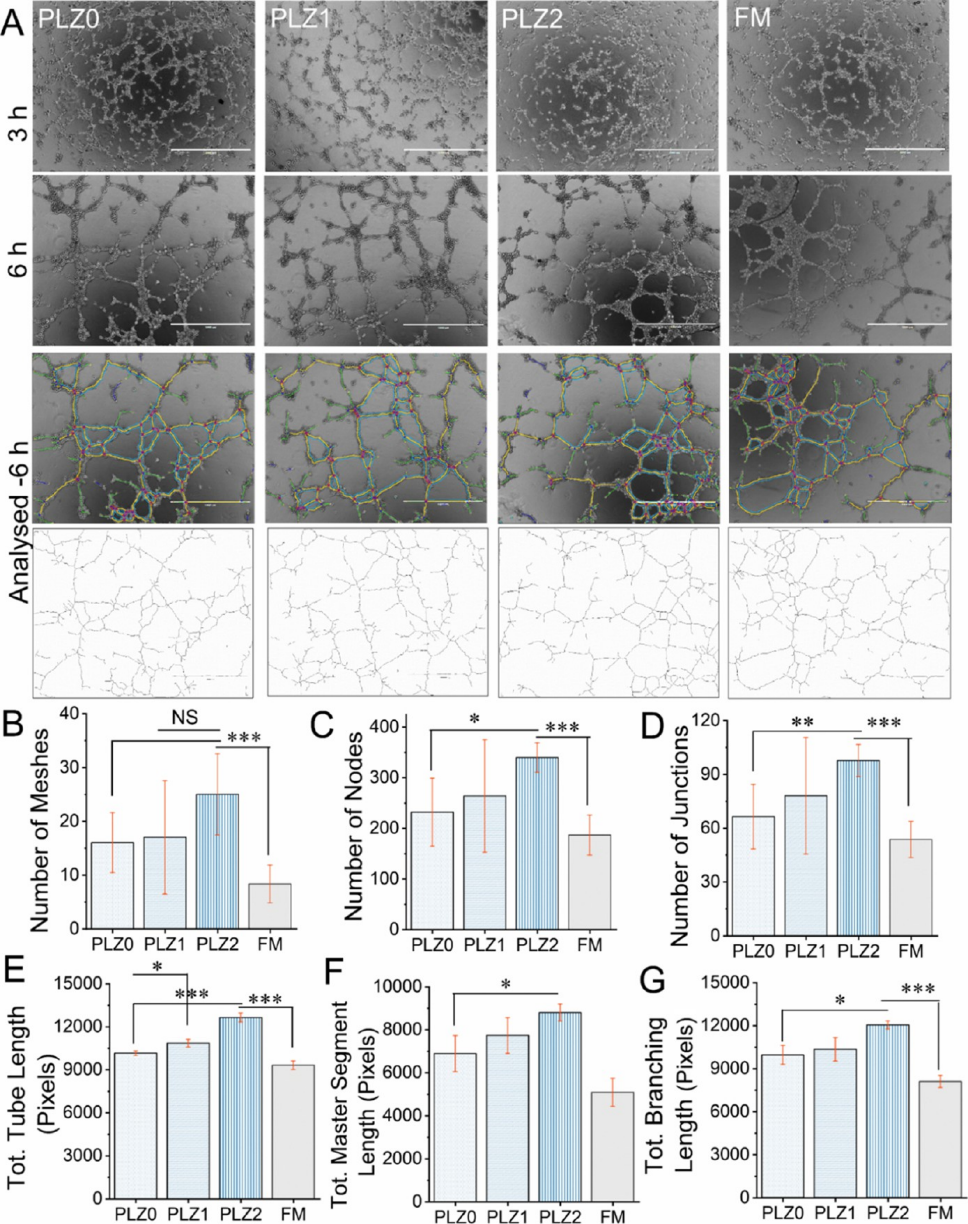
In vitro angiogenic potential of the extracted medium from the scaffolds and their effects on tube formation. (A) The formation of capillary network morphologies by HUVECs was observed and photographed using an inverted microscope at 3 and 6 h. Scale bar = 1000 μm. Bottom: respective images at 6 h were used to analyze tube formation using the angiogenesis plugin from ImageJ software. Measurement of different parameters of tube forming ability, including numbers of meshes (B), the number of nodes (C), the number of junctions (D), total tube length (E), total master segments length (F), and total branching length (G). One-way ANOVA with Tukey’s post hoc test was used to perform statistical significance between the groups, and the data were expressed as the mean ± SD; *n* = 3 per group (where NS = no significance, *p < 0.05, **p < 0.01, and ***p < 0.001).

**Figure 10:**
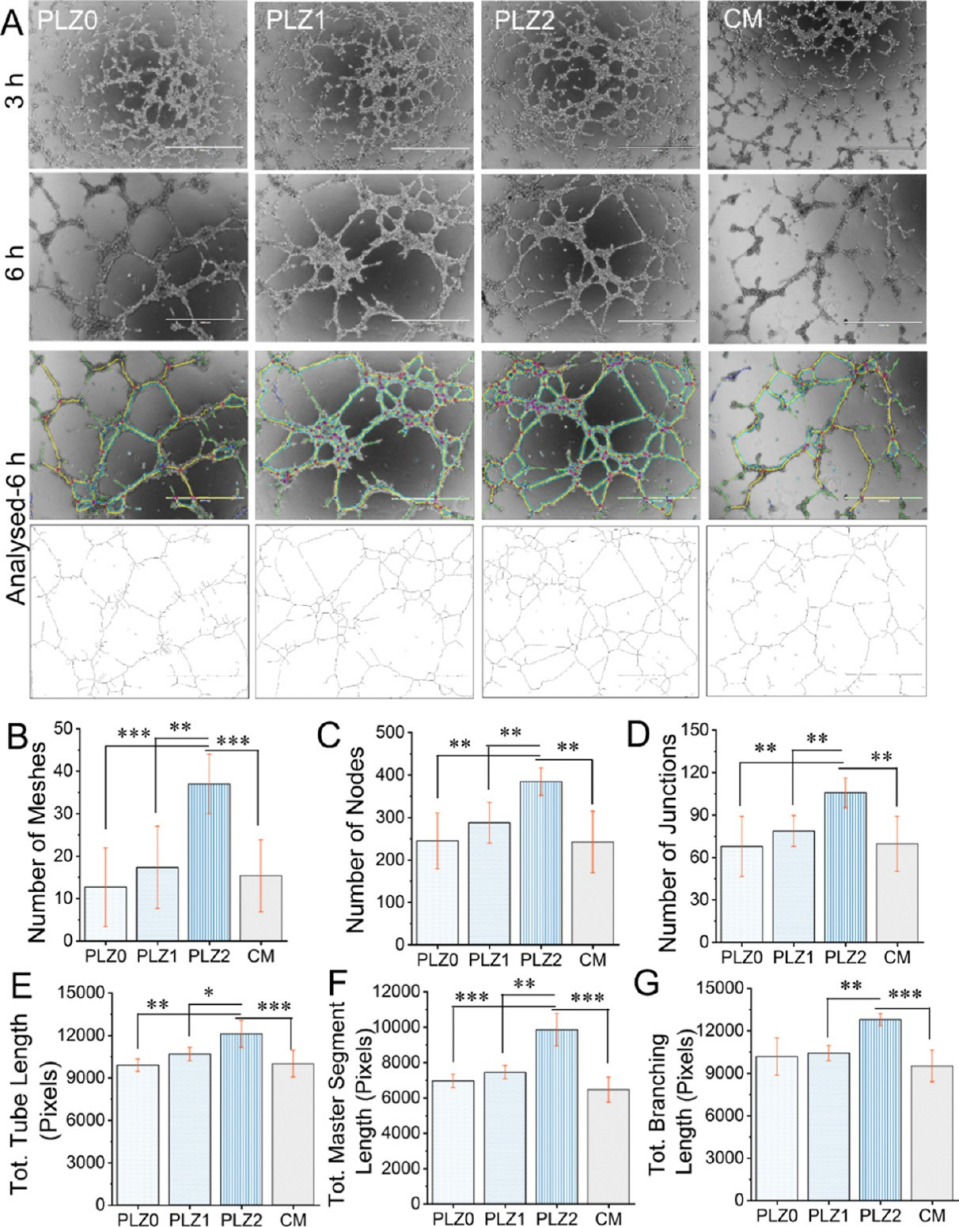
In vitro angiogenic potential of CCM on tube formation. (A) The formation of capillary network-like structures by HUVEC was observed and photographed using an inverted microscope at 3 and 6 h. Bottom: respective images at 6 h were used to perform analysis with the angiogenesis plugin for ImageJ software. Different parameters were reported as a measurement of tube forming ability, such as the number of meshes (B), the number of nodes (C), the number of junctions (D), total tube length (E), total master segments length (F), and the total branching length (G). Scale bar = 1000 μm. One-way ANOVA with Tukey’s post hoc test was used to perform statistical significance between the groups, and the data were expressed as the mean ± SD; *n* = 3 per group (where *p < 0.05, **p < 0.01, and ***p < 0.001).

### 3.9 Angiogenetic Differentiation of HUVECs In Vitro

The assessment of angiogenesis and formation of vasculature morphology by HUVECs was examined through immunocytochemistry (Figure 11) using sensitive and specific markers (CD31 and VE-cadherin protein) for vascular differentiation, which controls the cell–cell cohesion and organization of the intracellular junction. The immunofluorescence images in Figures 11A,B, S12, and S13 showed the expression of the vascular endothelial tight junction molecule, CD31, expressed by the HUVECs cultured on extracts of different scaffolds and the CCM, respectively. HUVECs proliferated greatly and formed a monolayer with increased number of tight junctions at day 7. Compared to the extract groups, a significant increase in the density and degree of neovascularization was observed in the CCM of the different groups. It can be seen that the majority of the cells expressed CD31 protein markers, with a red circle around the entire periphery of the nuclei interconnected between the cells, representing the vascular differentiation of HUVECs. Remarkably, the Zn-based scaffolds (PLZ1 and PLZ2) promoted larger vessel formation and continuous vascular networks, indicating an enhanced angiogenesis. This effect is attributed to the controlled release of Zn^2+^, which upregulates CD31 expression and supports vascular growth in diabetic wound healing.^59^ Moreover, Zn^2+^ and VEGF in the CCM could promote a variety of signaling mechanisms for guiding cytoskeletal reorganization along with cellular functions like proliferation, migration, capillary-like networks/tubule formation, and vascularization of HUVECs.^16^ The quantitative analysis of the respective fluorescence images from Figure 11E,F shows an up-regulated expression level of CD31, which is predominantly higher. The values for PLZ0, PLZ1, and PLZ2 were statistically significant (*p* < 0.001) compared with the control groups. Meanwhile, the value for PLZ1 and PLZ2 was also statistically significant (*p* < 0.001) compared with PLZ0. Notably, the expression of CD31 continues to increase in the order of PLZ0< PLZ1< PLZ2 in both the extracted medium and CCM. The expression of CD31 increased along with cell proliferation, and many tight junction proteins were noticed throughout the cell membrane. The HUVECs grown with CM (HDFn-grown medium on TCP) showed a higher expression level of CD31 than HUVECs grown in TCP with FM. The HDFn produces bFGF and other angiogenic factors that influence the cellular activity of another cell type, like HUVECs. The growth factors produced by HDFn could promote HUVECs to spread during the angiogenesis process, supporting cell–cell interactions to maintain a capillary-like structure, which may perhaps be connected to their ability to produce new ECM. Grajar et al. reported that fibroblasts stimulate endothelial cells to form mature capillary networks. These fibroblast-mediated capillary morphologies are dependent on matrix metalloproteinase production, where the proteinases are responsible for wound healing, angiogenesis, and tissue remodeling.^60^

**Figure 11:**
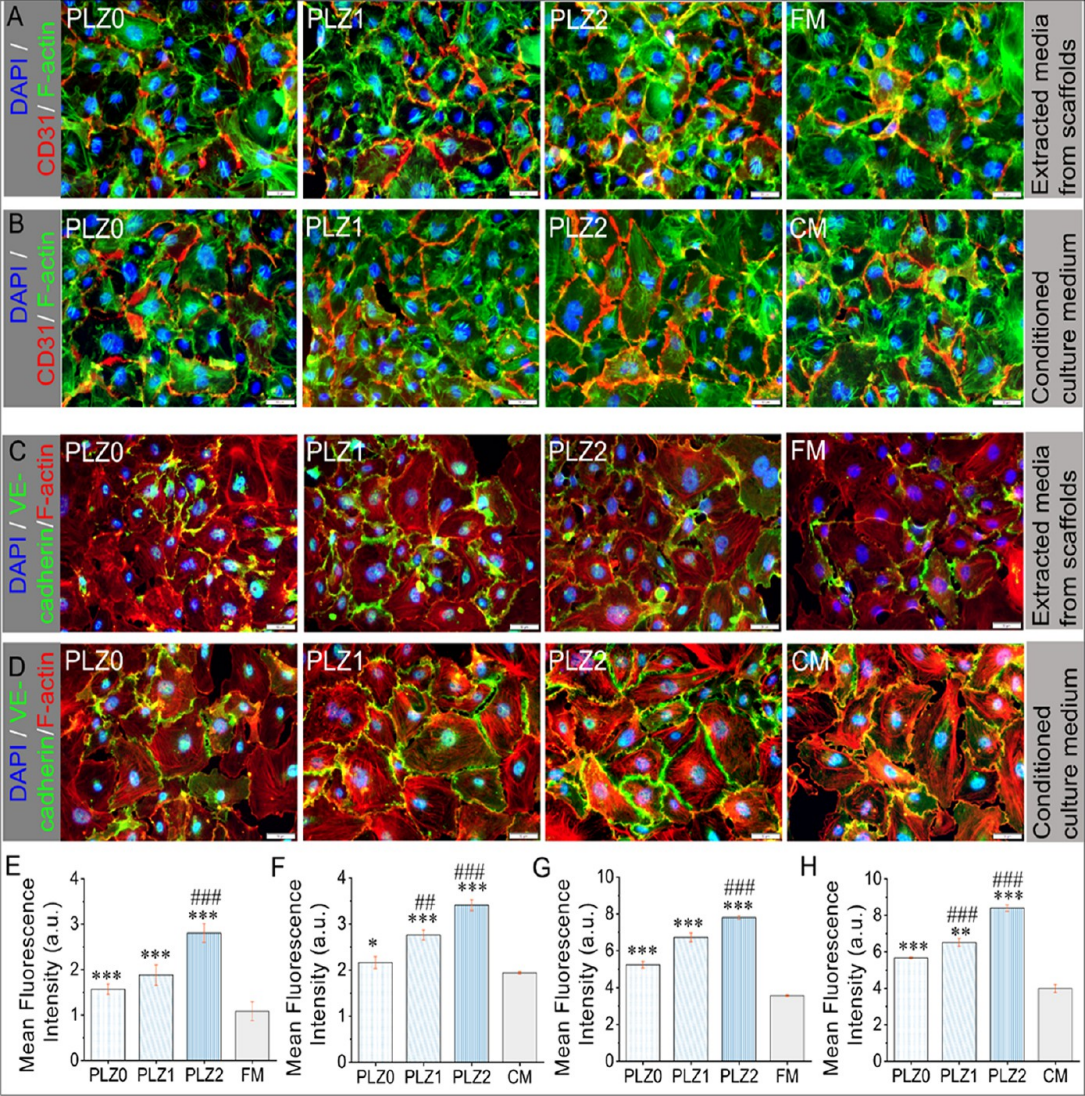
In vitro angiogenic potential of the extracted medium from scaffolds and CCM on vasculature formation. Immunofluorescence images displaying the endothelial-junction-associated protein markers, CD31 (red) and VE-cadherin (green), by the HUVECs cultured under-extracted medium (A and C) and CCM (B and D) from different scaffolds for 7 days. F-actin in A and B was labeled with ActinGreen 488 ReadyProbes Reagents (AlexaFluor 488 phalloidin). While for C and D, F-actin was labeled with ActinRed 555 ReadyProbes Reagents (Rhodamine phalloidin). Scale bar = 50 μm. Quantification of fluorescence intensity of the expression of CD31 and VE-cadherin of respective immunofluorescence images from the HUVECs grown in extracted medium (E and G) and CCM (F and H), respectively, was analyzed by the ImageJ software. Statistical analysis was performed using the one-way ANOVA with Tukey’s post hoc test, and the data were expressed as the mean ± SD; *n* = 3 per group (where *p < 0.05, **p < 0.01, and ***p < 0.001 compared with controls (FM and CM), while ##*p* < 0.01 and ###*p* < 0.001 compared with PLZ0).

Moreover, vascular endothelial cell-to-cell adherent junctions were visualized through immunofluorescence staining of the VE-cadherin protein, as shown in Figures 11C,D, S14, and S15, expressed by HUVECs cultured on extracted medium and in a CCM, respectively. The cells cultured in FM and CM were used as a control for each group. The expression of VE-cadherin was higher and circulated across the cell membrane, confirming the formation of interconnected and organized intercellular junctions in PLZ1 and PLZ2 (both on the extract and CCM). In the PLZ0 and FM, expression of VE-cadherin was less, intermittent, and showed frequent gaps along the cell–cell borders (Figure 11C). In contrast, staining and expression of VE-cadherin were a bit strong and continuously distributed around the periphery of cells in PLZ0 and CM (Figure 11D). There was a remarkable increment in the interconnectivity of the structure formed on the CCM of PLZ2 compared to other groups. Low expressions and altered distribution of VE-cadherin indicate that the endothelial barrier is interrupted, causing vascular permeability.^61^ Further, growth factors such as VEGF (VEGFR1 and VEGFR2) activate the endothelial cells’ proliferation, migration, and permeability, which is necessary for angiogenesis, vascular homeostasis, and different diseases to facilitate VE-cadherin internalization.^6,62^ The quantitative analysis of corresponding immunofluorescence images (Figure 11G,H) shows VE-cadherin expression, which was significantly higher (*p* < 0.01 to *p* < 0.001) in the PLZ1 and PLZ2. The level of expression of VE-cadherin gradually increased with cell proliferation and maturation, and many cell–cell adherent junction proteins were found throughout the cell membrane. These results indicate that extracts of PLZ1 and PLZ2 positively induced HUVECs to undergo neovascularization and tube formation. Zn^2+^ and growth factors in the CCM significantly enhanced the cellular activity. The integrated strategy on the modulation of the coculture microenvironment could specify an effective way to regulate and speed up wound healing and tissue regeneration precisely.

### 3.10 Western Blot Analysis and the Role of Zn^2+^ in Wound Healing

To elucidate the underlying mechanism of Zn^2+^ released from the scaffolds in promoting wound healing, we investigated its impact on key cellular processes using Western blot analysis across three different cell types: macrophages (RAW264.7), fibroblasts (HDFn), and endothelial cells (HUVECs). Our results provide strong evidence that Zn^2+^ regulates a multiphase, multicellular response essential for tissue repair. The expression of iNOS, TNF-α, and Arg1 in RAW264.7 cells (Figure 12A), with cells cultured on PLZ2 exhibiting significantly higher protein levels (*p* < 0.001) compared to other scaffold groups. These findings align with immunomodulatory profiles shown in Figure 6, supporting zinc’s role in macrophage activation and phenotype transition. Furthermore, the upregulation of VEGF, α-SMA, and collagen III in HDFn cells (Figure 12B) indicates that the Zn^2+^-enriched microenvironment significantly enhanced fibroblast activity (*p* < 0.05), promoting angiogenesis and extracellular matrix remodeling. Zn^2+^ also played a pivotal role in maintaining the endothelial barrier integrity by regulating the expression and localization of key endothelial markers, CD31 and VE-cadherin, in HUVECs. The impact of Zn^2+^ on endothelial cells was further evident in the pro-angiogenic pathway stimulated by HDFn-conditioned medium (Figure 12C). The significant expression of these markers (*p* < 0.001) underscores the influence of Zn^2+^ on endothelial migration and elongation, supporting neovascularization and strengthening cell–cell junctions during tissue repair.

**Figure 12:**
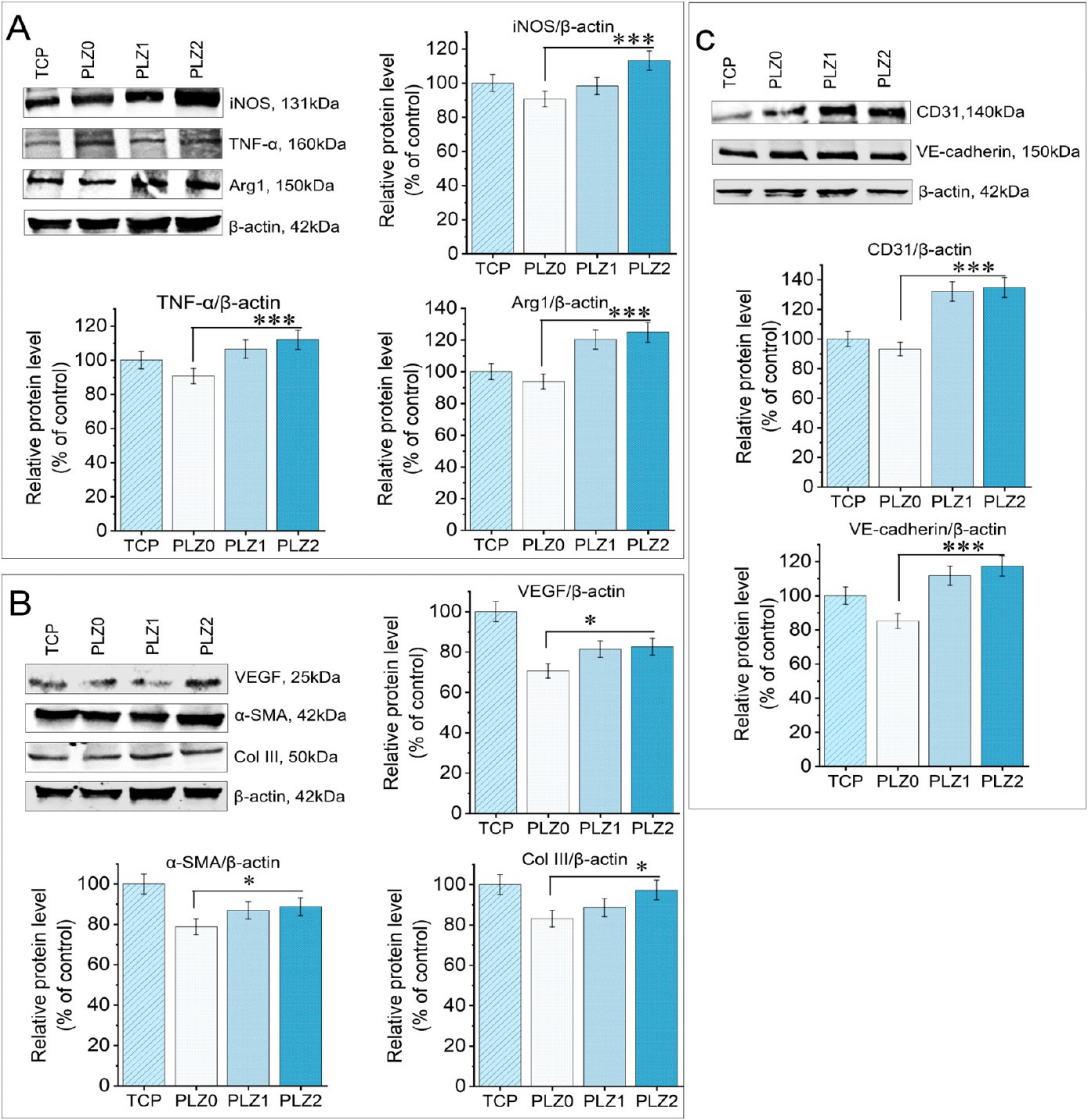
Western blot analysis and quantitative evaluation of protein expression. (A) Representative Western blot images and corresponding densitometric analysis of RAW264.7 macrophages, showing expression of iNOS, TNF-α, and Arg1. Cells cultured on PLZ2 exhibited significantly higher levels of these immunomodulatory proteins compared to other groups, indicating enhanced inflammatory activation and reparative phenotype transition. (B) Western blot analysis of HDFn demonstrating upregulation of VEGF, α-SMA, and Collagen III in zinc-enriched scaffolds, suggesting improved angiogenic signaling and extracellular matrix remodeling. (C) Western blot analysis of HUVECs in CCM, showing increased expression of CD31 and VE-cadherin, key markers of endothelial junction integrity and vascular stability. β-Actin served as the internal loading control for all blots. TCP was used as the baseline control for each group. Quantitative data are presented as mean ± SD (*n* = 3). Statistical significance was determined using one-way ANOVA followed by Tukey’s post hoc test (*p < 0.05 and ***p < 0.001).

Cellular Zn levels are tightly regulated by a network of Zn transporter proteins, with the SLC39 (ZIP) family mediating zinc influx and the SLC30 (ZnT) family facilitating zinc efflux to maintain Zn^2+^ homeostasis within the cytoplasm.^14^ Physiological levels of Zn^2+^ modulate NF-κB, MAPK/ERK, and STAT3 pathways, and regulate IL-1β and IL-6, and promote VEGF, EGF, and bFGF production, supporting angiogenesis and fibroblast proliferation.^63^ The homeostasis of Zn^2+^ regulates the polarization of RAW264.7 macrophage into M1 and M2 phenotypes, which helps coordinate inflammation resolution and ECM remodeling.^64^ Elevated iNOS and TNF-α levels with our scaffolds indicate activation of pro-inflammatory pathways during the early phase of wound healing, facilitating pathogen clearance and immune cell recruitment.^46^

Concurrent upregulation of Arg1 suggests a phenotypic shift toward an M2-like reparative state, promoting anti-inflammatory signaling and tissue regeneration. These findings support our scaffold’s role in balancing inflammation and repair, similar to what has been proposed in recent literature.^14^ In HDFn cells, Zn^2+^ significantly upregulated the expression of VEGF, α-SMA, and Col III. VEGF upregulation supports angiogenesis^48^ whereas α-SMA indicates fibroblast differentiation into myofibroblast, which is critical for wound contractions.^38^

Zinc homeostasis enhances cellular proliferation by stimulating angiogenesis and facilitating Collagen III deposition, thereby supporting skin regeneration and wound healing. Cytosolic labile Zn^2+^ restores these processes by activating FGF2/FGFR and VEGFR/VEGFR-2 signaling, promoting fibroblast proliferation, endothelial angiogenesis, and coordinated fibroblast–endothelial interactions.^62,65^ HUVECs exposed with the HDFn-conditioned medium exhibited significant upregulation of CD31 and VE-cadherin, essential markers for neovascularization and strengthening cell–cell junctions during tissue remodeling. Additionally, the pro-angiogenic signaling observed with HDFn-conditioned medium suggested synergistic interactions between fibroblasts and endothelial cells. The Zn^2+^ induces a conformational change in T2-TrpRS, a truncated form generated from tryptophanyl-tRNA synthetase, enhancing its binding to VE-cadherin and enabling the regulation of angiogenesis.^66^ Collectively, these findings reflect the importance of sustained release of Zn^2+^ from Zn nanoparticle incorporated nanofibrous scaffolds to restore the angiogenic–fibrogenic balance necessary for enhancing wound closure and tissue regeneration.

## 4 Conclusions

In summary, we fabricated biodegradable PLGA fibrous scaffolds incorporating Zn NP by using electrospinning. The scaffolds were physicochemically characterized and studied for their bioactivity with HDFn, HUVECs, and macrophages. The in vitro HDFn-laden scaffolds showed enhanced viability, proliferation, and differentiation into activated fibroblasts and myofibroblasts, as shown by vimentin and α-SMA expression. The scaffolds facilitated macrophage polarization from the pro-inflammatory M1 phenotype to the reparative M2 phenotype, confirmed by iNOS and Arg1 expression. Similarly, cells cultured on Zn-containing scaffolds exhibited significantly higher levels of these immunomodulatory proteins compared with controls, indicating enhanced inflammatory activation alongside a transition toward a reparative state. Moreover, in an indirect HDFn–HUVEC coculture, the CCM enhanced the growth, migration, and tube formation of HUVECs. HDFn proliferation and differentiation were accompanied by the secretion of VEGF and bFGF, which, together with Zn^2+^, enhanced HUVEC metabolic activity and induced neovascularization, evidenced by CD31 and VE-cadherin upregulation. Overall, the coculture system favored cell-to-cell communication, thereby supporting critical cellular and molecular processes. Collectively, these findings emphasized the importance of extracellular Zn homeostasis within the microenvironment in regulating HUVECs’ function and promoting angiogenesis, vascularization, and tissue regeneration.

## Supplementary Material



## Notes

SEM-EDS mapping of PLZ2 fibers; colorimetric Zn^2+^ detection assay and optical images; detail characterization of fibrous scaffolds using XRD, FTIR, TGA, and DSC; mechanical test, water contact angle, and degradation; cell viability and live/dead staining on different fibrous scaffolds; scaffolds effect on cell attachment and morphology; fibroblast immunocytochemistry; macrophage polarization markers; HUVECs live/dead staining in conditioned medium; endothelial-junction-associated proteins CD31; and VE-cadherin (PDF)
