# Supplementary material for: Zinc-Releasing Fibrous Scaffolds Modulate Fibroblast, Endothelial, and Macrophage Interactions for Vascularized Tissue Engineering
Source: ACS Appl Mater Interfaces. 2026 Jan 6;18(2):3477–98. doi: 10.1021/acsami.5c16589 (PMC12828720; doi:10.1021/acsami.5c16589)
Supplement: Supplementary file 1 [file am5c16589_si_001.pdf]

Supporting Information

Zinc-Releasing Fibrous Scaffolds Modulate Fibroblast, Endothelial, and Macrophage Interactions for Vascularized Tissue Engineering

Sita Shrestha [a†], Bishnu Kumar Shrestha [a†], Reedwan Bin Zafar Auniq [b], Niranjan Parajuli [a], Salil Desai [c], Narayan Bhattarai [a] *,

[a] Department of Chemical, Biological, and Bioengineering, North Carolina A&T State University, Greensboro, North Carolina 27411, United States

[b] Department of Nanoengineering, Joint School of Nanoscience and Nanoengineering, North Carolina A&T State University, Greensboro, NC 27401, United States

[c] Department of Industrial and Systems Engineering, North Carolina A&T State University, Greensboro, North Carolina 27411, United States

*Correspondence to: Narayan Bhattarai, Department of Chemical, Biological, and Bioengineering, North Carolina A&T State University, Greensboro, NC, 27411, USA

Email: nbhattar@ncat.edu

† Equal Contribution

Fiber Morphology and Colorimetric Zinc Detection Assay

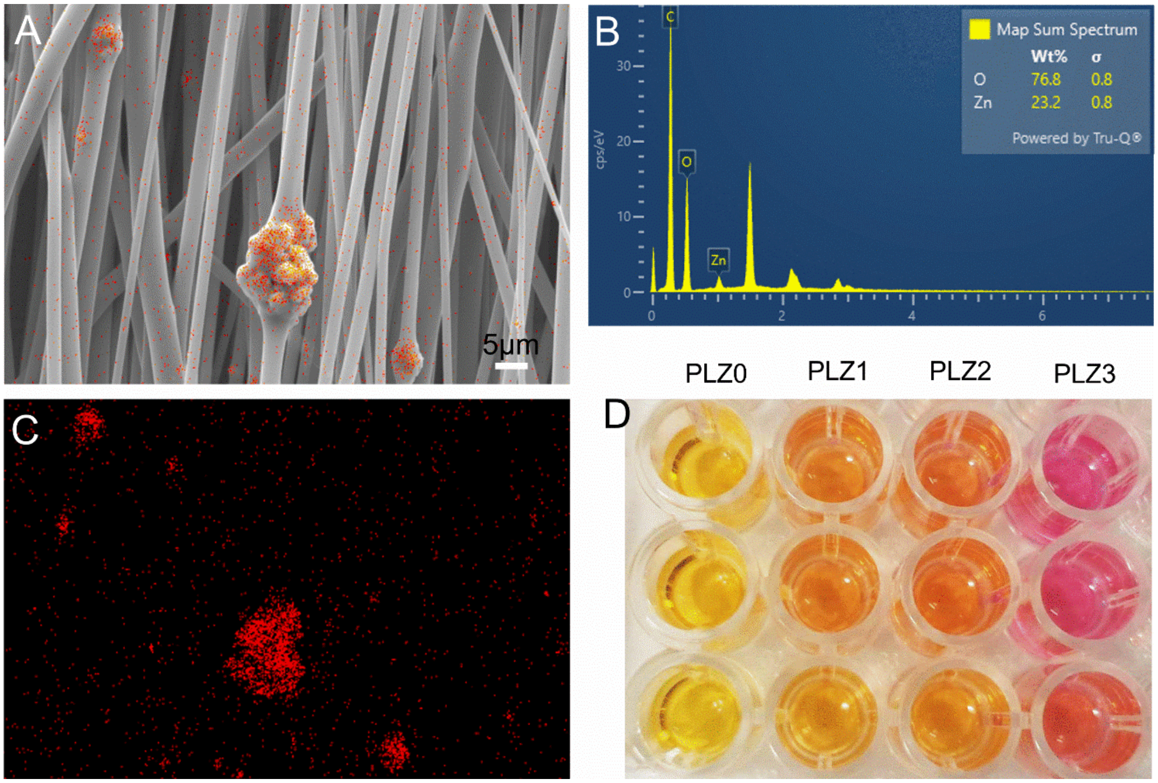

Figure S1. (A-C) SEM-EDS mapping image of PLZ3 fibers. Scale bars = 5 μm (D) Colorimetric Zn^{2+} detection assay, Optical images of colorimetric Zn^{2+} detection on cell-cultured medium of different fibrous scaffolds.

Analysis of Physicochemical Properties

Experimental Section: The crystallinity of each material was also examined using X-ray diffraction (Rigaku MiniFlex XRD with D/teX Ultra2 detector) with a Soller Slit 5.0' (Variable and Fixed Slit system). Brag-Brentano (BB) Powder with a 2θ range of (5 – 60) ° and step size 0.01 at a scan rate of 10 °/min was applied for measurement. The Fourier transmission infrared (FTIR) spectra of the fibrous mesh were recorded by an FTIR spectrometer (Agilent 670, Santa Clara, CA, USA). TA Q200 Thermogravimetry Analysis (TGA) was conducted to observe the mass loss, and was measured using a TGA instrument (TGA 5500, USA). The TGA analysis was carried out by heating the fibrous sample from room temperature (RT) to 700 °C, at a heat rate of 10 °C/min in a

nitrogen environment, with a gas flow rate of 25 ml/min. Differential Scanning Calorimetry (DSC) Analysis was performed to evaluate the temperature and heat flow of the different fibrous mesh samples. The samples were heated from RT to 450 °C at a heating rate of 10 °C/min in a nitrogen environment, with a gas flow of 50ml/min.

The mechanical properties of the fibrous mesh (customized template constructed from cardstock; 25×18 mm) were examined using a TA. XT Plus Texture Analyzer (Hamilton, MA). A (n=3) fibrous mesh (16 x 9 mm) was firmly affixed to the template with double-sided tape at both ends before fibrous mesh testing. A digital micrometer was used to measure the thickness of the meshes, and the average value was used. The affixed fibrous mesh was placed between the pneumatic jaw gripping, and before testing, both sides of the template were cut. The fibrous mesh was stretched until failure with a 500 N load cell and a 3 mm/min set displacement. After each run, a load (N) versus extension (mm) curve was generated, and the load values were divided by the cross-sectional area (mm^2) to obtain the stress values (MPa). The strain values were calculated by dividing the change in length by the initial length. Stress – strain curves were plotted using Origin Pro, and mechanical strength was measured.

Further, an enzymatic biodegradation test was performed for different scaffolds. The dry scaffolds were immersed in PBS containing 10mg/mL lysozyme (Millipore Sigma) for 1, 3, and 7 days. The concentration of the enzyme was based on the ASTM standard (F-1635-95). After the various time intervals, the scaffolds were washed with DI water several times and lyophilized. SEM was used to examine the morphology of lyophilized scaffolds. The hydrophilicity of the fibrous scaffolds was determined through the static contact angle measurement using the sessile drop method (KRUSS drop shape analyzer, DSA25E, Germany) at RT. The optical image of each fibrous

scaffold absorbing water droplet was taken at 30 seconds after the droplets' deposition on the scaffold's surface (n = 3).

Results and Discussion

To confirm the presence of Zn particles embedded in the fibrous scaffold, the scaffold's crystallinity was examined **(Figure 2A).** Pure PLGA showed no crystalline peaks, indicating an amorphous structure. However, diffraction peaks at 2θ = 36.2°, 38.7°, and 43.3° are assigned to the (002), (100), and (101) crystalline planes of Zn particles, respectively, and were observed in PLZ1 and PLZ2. It has also been shown that a peak at 56.2° corresponds to the (102) plane of Zn particles, suggesting that the hexagonal shape of most particles is encapsulated, with some were exposed on the surface of the fibers. [1]

Figure 2B shows the FTIR spectra of pure PLGA fibers after treatment with TFE. The peak positions are consistent with the reported literature. [2] It was observed that the peaks at 1,752 cm^{-1} are assigned to the ester linkage of the C=O group, but asymmetric stretching of C-O-C was confirmed by the intense typical peaks in the range of 1187 to 1454 cm^{-1}. Importantly, peaks were positioned around 2853 cm^{-1} to 3004 cm^{-1}, confirming the stretching mode of methyl and methylene groups of the copolymeric unit of lactic acid and glycolic acid. However, no significant changes in peaks position were observed after encapsulation of Zn particles in the fibers. The polymeric composite showed an increase in peak intensity because of the surface-enhanced infrared absorption. The Zn particles exhibit the localized surface plasmon resonance, and the absorption of infrared enhances the vibrational and stretching signals of the surrounding PLGA polymeric composite. [3]

To evaluate the thermal decomposition of the three different scaffolds (PLZ0, PLZ1, and PLZ2) as a function of temperature, **Figure 2C** shows the variation in apparent weight loss curves of the

scaffolds. The PLZ0 began to lose its weight at a temperature from 297°C (onset temperature) to 366°C (endset temperature). During this range, thermal oxidation, carbonization of polymeric hydrocarbons, and decomposition of alkane groups took place. Beyond this temperature, no residual mass remained, indicating that the decomposition of polymeric units occurred through evaporation. The percentage weight loss of the fibrous meshes reached up to 97.5% at 366°C. Afterward, no residual weight was present beyond 470°C. In contrast, we observed that both the onset (257°C) and endset (307°C) temperatures of PLZ1 and PLZ2 were lower than those of PLZ0, which is a notable difference. The Zn particles in the polymeric fibers enhance thermal conductivity, enabling the efficient pathway of heat spread throughout the composite. Consequently, the polymeric composites heat more evenly via thermal transformation and begin to decompose earlier, indicating lower onset and endset temperatures. It is noteworthy that the encapsulation of metallic particles in fibers may induce thermal-catalytic and thermo-oxidative effects that also accelerate the decomposition of the polymer chains at lower temperatures. [4] The residual weight, consisting of Zn particles, metallic oxides, and inorganic ashes of about ~0.6% in PLZ1 and ~1% in PLZ2, persisted beyond 570°C (inset **Figure 2C)**, confirming that PLZ2 contains a higher wt% of Zn particles than PLZ1.

The thermal behavior of the fibrous scaffolds was evaluated using DSC, as depicted in **Figure 2D**. No marked phase changes were observed among the polymeric scaffolds in the glass transition temperature (T_g), which was measured at 55°C, suggesting a complete amorphous nature of the polymer. But release of excess amount of free energy/heat was observed on PLZ0 at 278°C as an exothermic process due to the conformational energy as a lower energy configuration or the combustion reaction in polymeric materials. But a uniform heat flow was observed on Zn encapsulated scaffolds. The endothermic process was observed at 347°C for PLZ0, confirming the

melting of PLGA, which is sharply changed for PLZ1 and PLZ2 around 308°C. Indeed, the embedded of Zn particles in polymer increases the thermal absorption, which causes the lower latent heat fusion of the composite.[5] Thus, the PLZ1 and PLZ2 need less heat energy to melt compared to the PLZ0.

Figure S2 shows the ultimate tensile strength (UTS) of the fibrous scaffolds. The UTS was in the increasing order from PLZ0 to PLZ2 (for example, 0.7 MPa for PLZ0 < 1.3 MPa for PLZ1 < and 1.42 MPa for PLZ2). The higher UTS of PLZ1 and PLZ2 confirms the encapsulation or embedding of Zn particles uniformly distributed within the fibrous scaffolds. Importantly, the Zn particles act as a filler in electrospun fibers, reinforcing the formation of composite materials. [6] Notably, the particles in the fibrous exhibit interfacial shear strength, which significantly increases toughness and mechanical strength.[7] Furthermore, the particles take up some mechanical load from the polymeric chain, which increases the stiffness of the fibers, confirmed by the notable improvement of the Young's modulus. In addition, it might be possible that the particles in the polymeric matrix enhance the conductivity of the electrospun solution, resulting in highly aligned fiber orientation, exhibiting greater tensile strength. Such a load-bearing capacity of the scaffolds could be a good choice in tissue engineering applications and indeed for wound healing applications.

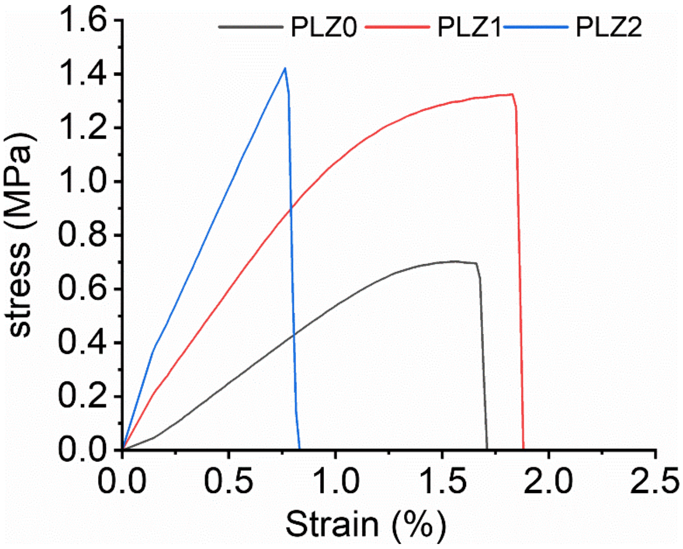

Figure S2. Analysis of Tensile mechanical properties for fibrous scaffolds. Representative set of stress – strain curves for PLZ0, PLZ1, and PLZ2 fibrous scaffolds.

The surface WCA of each fibrous scaffold was measured, and optical images are depicted in **Figure S3**. The Zn particles in fibrous scaffolds showed more hydrophilic properties with WCA of □ 80.3˚ compared to those without Zn particles (WCA > 107.8˚). The particles exposed on the surface of the fiber (shown in the TEM image, Figure 1) are susceptible to forming their oxide. These oxides react with water through hydrogen bonding and lower the WCA of the fibrous scaffold. [8] Notably, such property in the scaffolds supports cell and substrate interactions between the interface. The hydrophilicity of biomaterials enhances frequent remodeling of natural ECM to increase cell growth by transporting nutrients for cell attachment. [9]

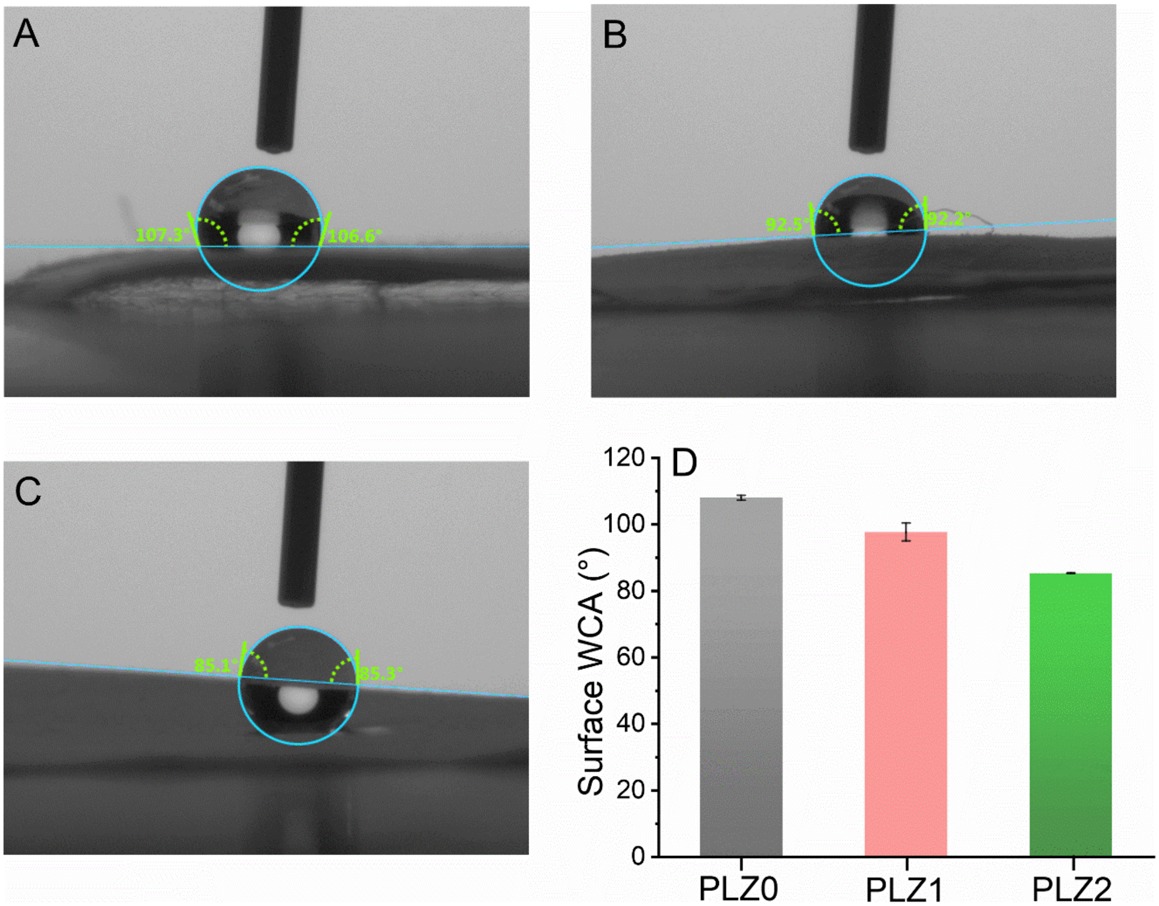

Figure S3. Water contact angle measurement of fibrous scaffolds. Optical images of surface water contact angle of PLZ0, PLZ1, and PLZ2 (A-C). Quantitative analysis of the water contact angle of each fibrous scaffold (D), (n = 3).

SEM images **(Figure S4)** illustrate in vitro enzymatic biodegradation of various fibrous scaffolds in the presence of lysozyme. Both composite scaffolds (PLZ1 and PLZ2) exhibited good biodegradation properties over time. After one week of enzymatic exposure, the macromolecular network of the regular and relatively uniform polymeric fibers was gradually broken down into discontinuous fibers of varying shapes and sizes, randomly oriented within increased surface roughness compared to their original morphology (SEM images in Figure 1). The notable changes in surface texture are attributed to the enzymatic hydrolysis of PLGA, accelerating the breakdown of ester bonds of the copolymer, producing lactic and glycolic acids. [10] Importantly, enzymes in

physiological fluids facilitate scaffold degradation. Such properties of the composite scaffold enable the quantitative controlled release of Zn^{2+}. These results demonstrate the rapid enzymatic degradation of the scaffolds, highlighting their potential as promising biomaterials for constructing functional bioscaffolds in tissue engineering applications.

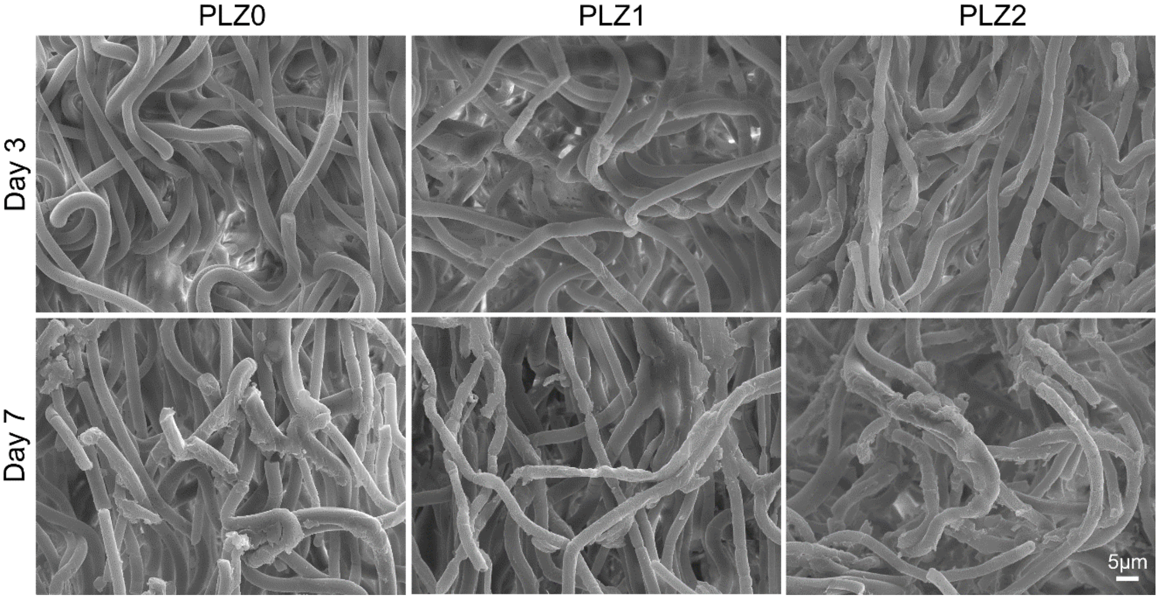

Figure S4. SEM images showing in vitro biodegradation of different fibrous scaffolds (PLZ0, PLZ1, and PLZ2) in 1X PBS (pH=7.4) containing 10mg/mL lysosomes after different days of immersion. Scale bar = 5 μm.

Cell viability, proliferation, attachments, and Immunocytochemistry (ICC) analysis on fibrous scaffolds

Primary Dermal Fibroblast Normal; Human Neonatal (HDFn, ATCC cell bank) and Human Umbilical Vein Endothelial Cells (HUVEC, CRL-1730™) were seeded on the scaffolds, separately attached to the circular glass coverslips (12 mm in diameter), and fixed at the bottom of 48-well plates. Before cell seeding, the prepared samples were sterilized under UV light for 3h and washed with 70% ethanol, followed by PBS (1X). The cells were seeded at a density of

1.5×10^4/well and incubated at 37°C in 5% CO_2 and 95% air atmosphere. The fresh cell culture medium was replenished every second day of the culture period for all the cell types. The cytotoxicity of the scaffolds was examined and determined with a live/dead assay kit (Perkin Elmer LLC Via AOPI Staining Solution; Fisher Scientific, USA) for both HDFn and HUVECs. Live cells were stained green and red for dead cells.

The viable cells' morphology, growth, and proliferation of HDFn and HUVECs were observed under a fluorescence microscope after staining the cells with Actin Green™ 488 Readyprobes™ reagent and DAPI. Cells were visualized under an Olympus I×83 microscope incorporated with Olympus Cell Sens Dimension software. Identification of heterogeneity of the HDFn was performed using the immunocytochemistry technique. The cells were incubated with primary antibodies: anti-Vimentin and anti-alpha-smooth muscle actin. The cells were labeled again with secondary antibodies, including goat anti-rabbit IgG H & L and goat anti-mouse IgG H & L. The nuclei were counterstained with DAPI for 10 min. Then, the Olympus I×83 microscope (Olympus) was used to visualize fluorescence images of stained cells. The fluorescence intensity of the cytoplasmic protein markers (vimentin and α-SMA) was expressed.

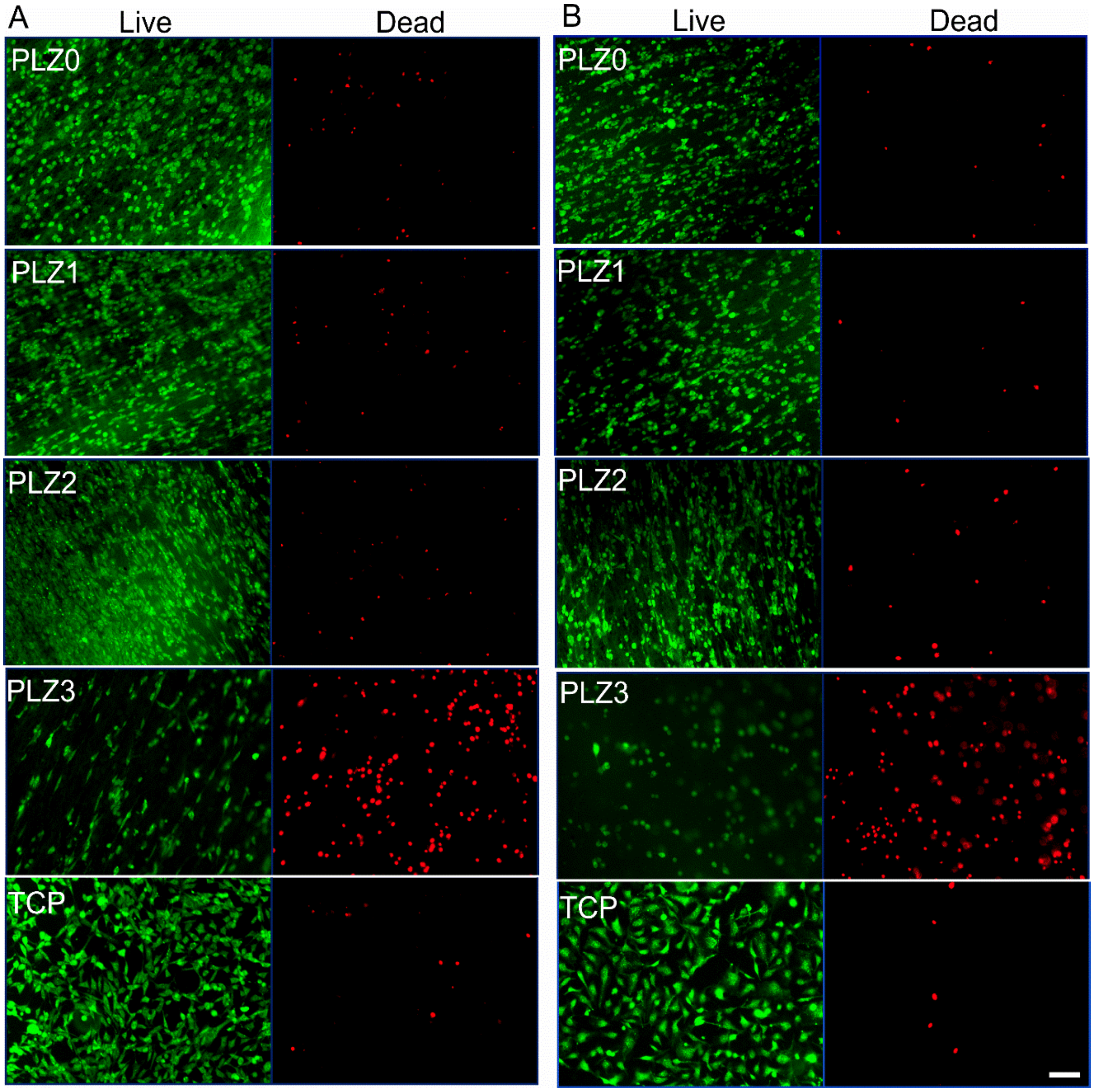

Figure S5. In vitro cell viability study via live /dead staining. (A) Fluorescence images for HDFn and (B) HUVECs seeded on different fibrous scaffolds at 5 days. Live cells (green color) and dead cells (red color) are stained using Perkin Elmer LLC Via Acridine Orange/ Propidium Iodide (AOPI) Staining Solution. Scale bar = 100 μm.

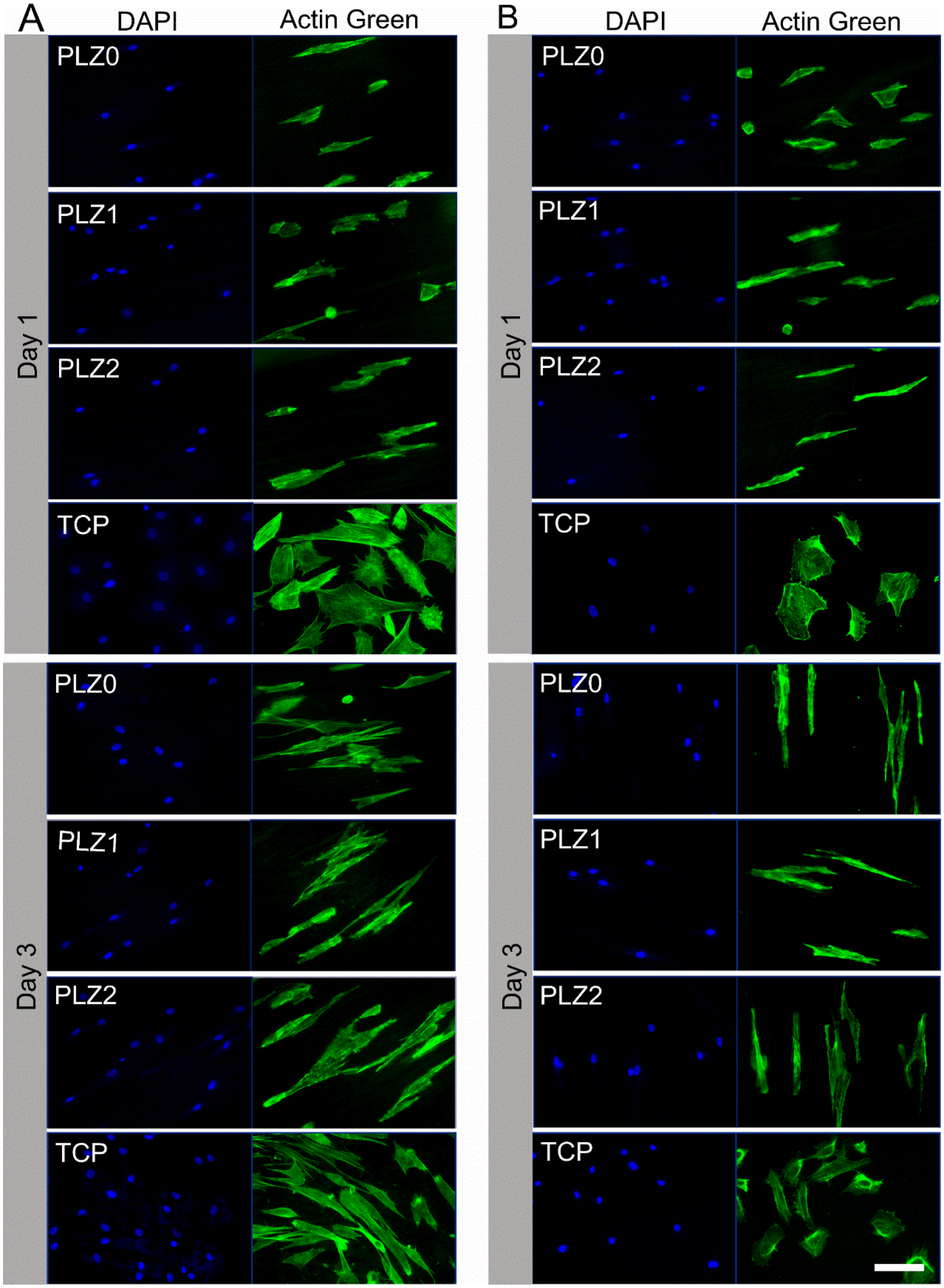

Figure S6. In vitro performance of the fibrous scaffolds on cell attachment and morphology. Fluorescence microscopy images of fibroblast cells (A) HDFn (B) HUVECs incubated with

different fibrous scaffolds on day 1 and day 3. Scale Bar = 50 μm. Staining showing the cytoskeleton with ActinGreen™ 488 (green) and the nuclei with DAPI (blue).

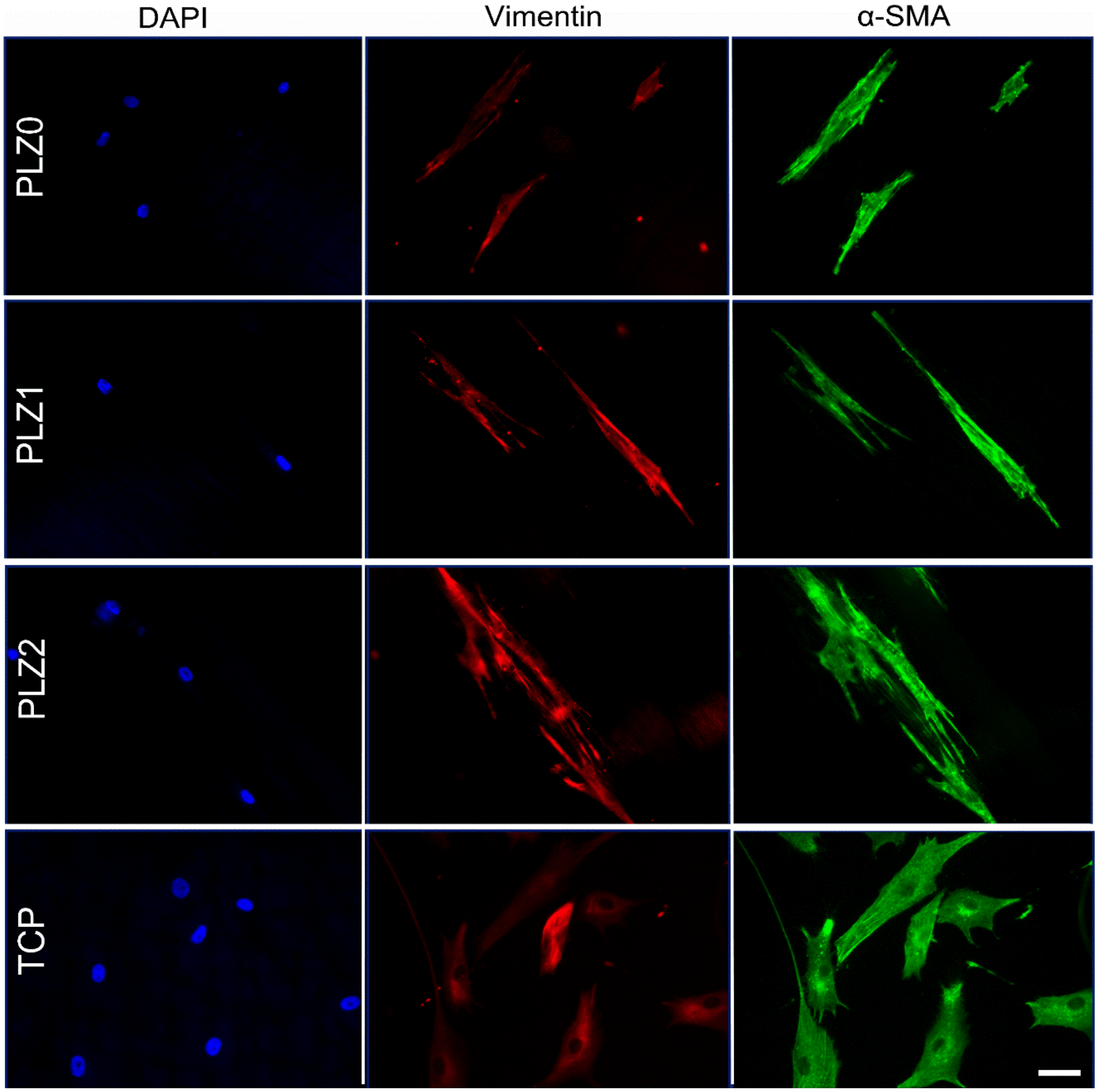

Figure S7. Immunocytochemistry of fibroblasts attached to the fibrous scaffolds showing the heterogeneity of HDFn at day 7. Cells were stained for Alpha (α)-Smooth Muscle Actin (green) and Vimentin (red), a characteristic protein expression. Nuclei were counterstained with DAPI (blue). Cells were visualized under an Olympus I×83 microscope. Scale bar = 50 μm.

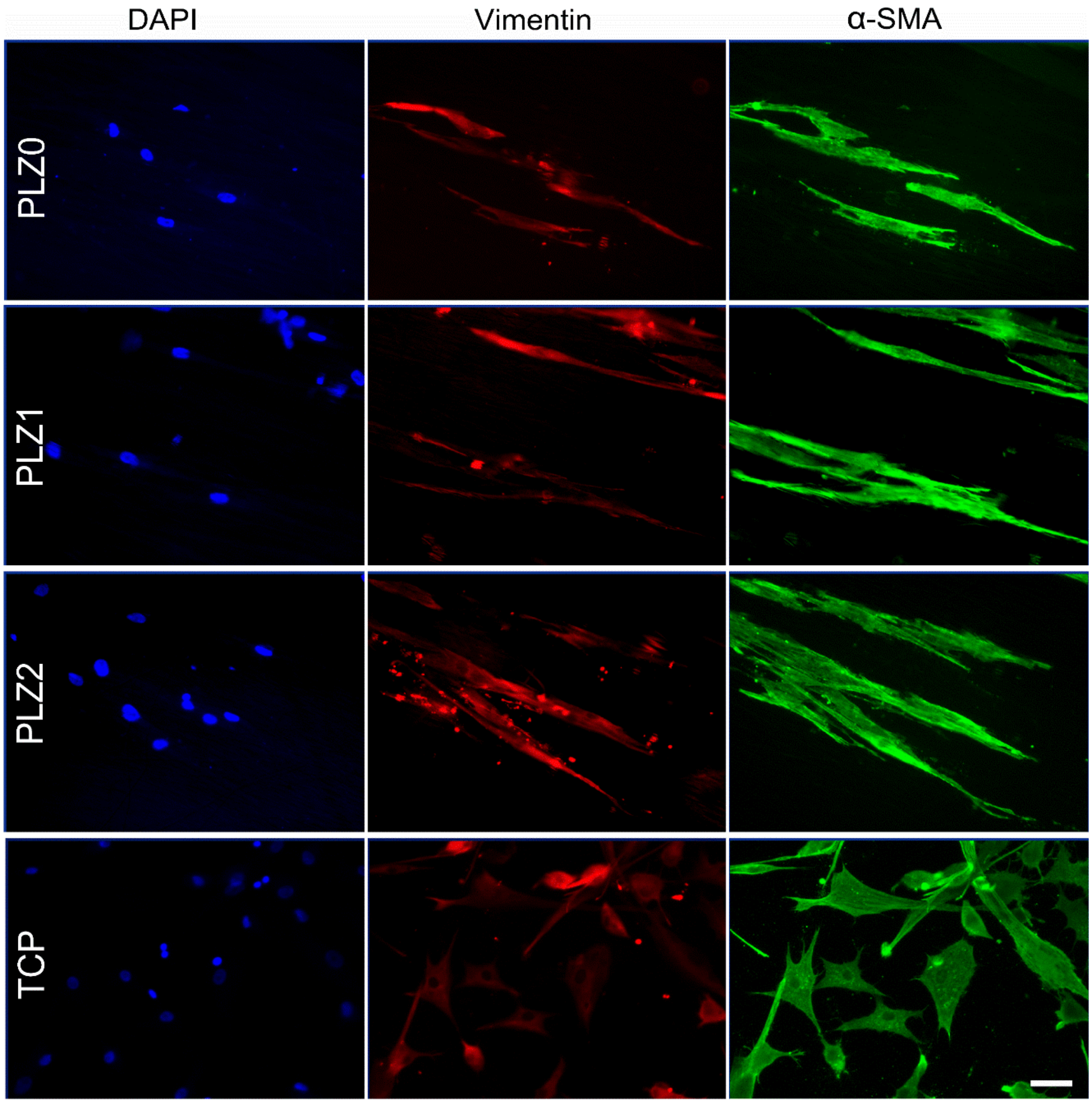

Figure S8. Immunocytochemistry of fibroblasts attached to the fibrous scaffolds showing the heterogeneity of HDFn at day 14. Cells were stained for Alpha (α)-Smooth Muscle Actin (green) and Vimentin (red), a characteristic protein expression. Nuclei were counterstained with DAPI (blue). Cells were visualized under an Olympus I×83 microscope. Scale bar = 50 μm.

In Vitro Macrophage Polarization on Fibrous Scaffolds

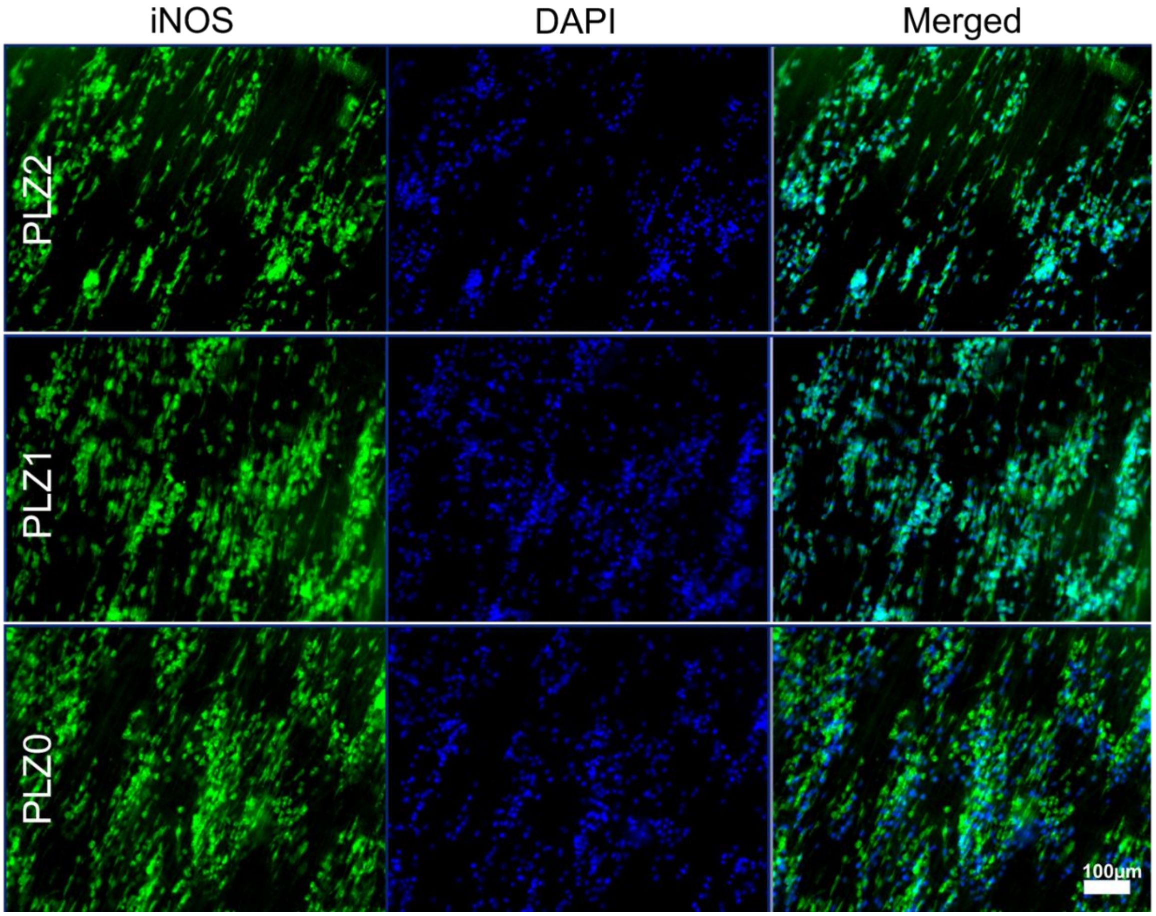

Figure S9. Immunofluorescence images of macrophage polarization markers – iNOS on different fibrous scaffolds after day 3. Scale bar = 100 µm.

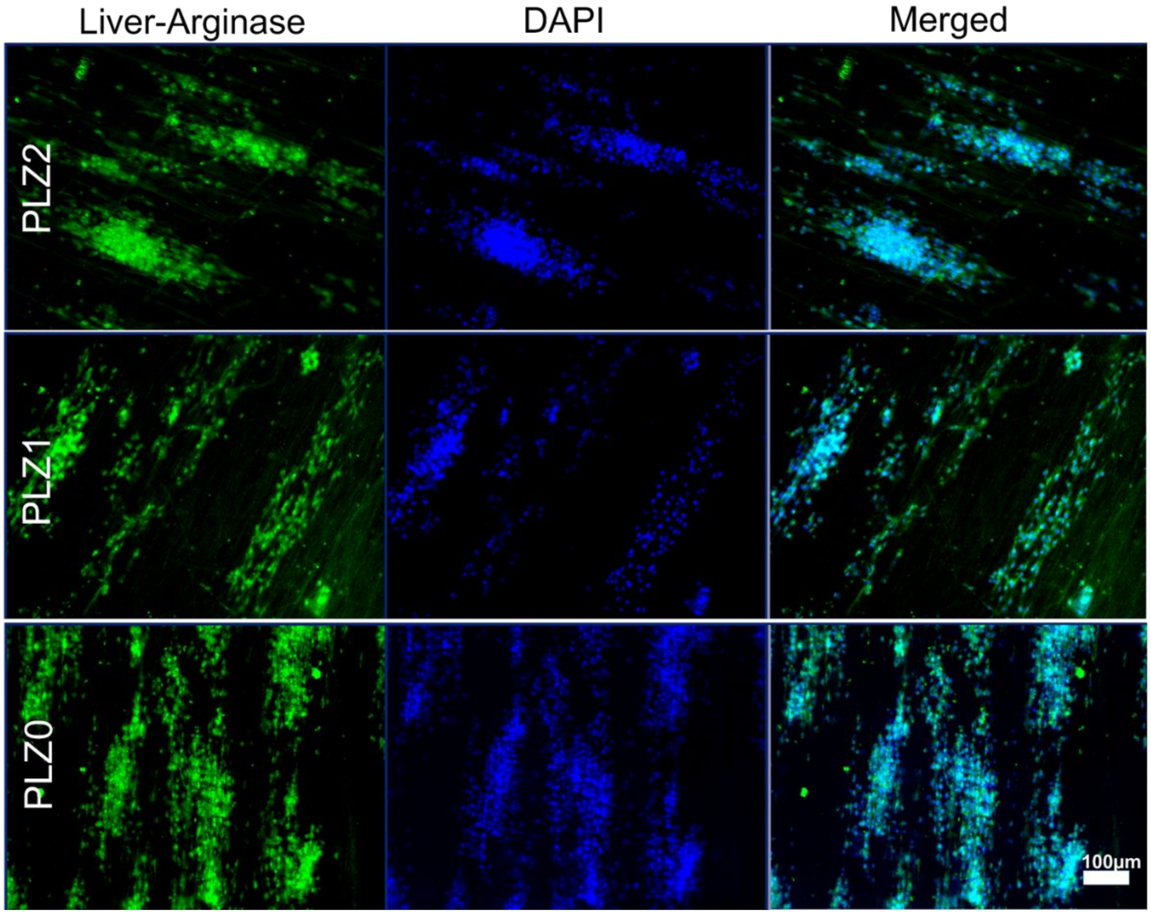

Figure S10. Immunofluorescence images of macrophage polarization markers – Agr1 on different fibrous scaffolds after day 3. Scale bar = 100 µm.

1. **Cell viability, proliferation, attachments, and Immunocytochemistry (ICC) analysis on the co-culture model under Conditioned Medium**

The survivability of the HUVECs cultured on indirect co-culture model on the Conditioned Medium from HDFn was examined with a live/dead assay kit (Perkin Elmer LLC Via AOPI Staining Solution; Fisher Scientific, USA), The live and dead cells stained in green and red color respectively were visualized and captured with an Olympus I×83 microscope incorporated with Olympus cell Sens Dimension software (Olympus Corporation, Shinjuku, Tokyo, Japan).

The morphology of the HUVECs grown on Conditioned medium was observed under a fluorescence microscope after staining the cells with Actin Green™ 488 Readyprobes™ reagent and DAPI. Cells were visualized under an Olympus I×83 microscope incorporated with Olympus Cell Sens Dimension software. The protein expression relevant to endothelial cell differentiation, VE-cadherin and CD31 immunostaining, was performed. Briefly, HUVECs were seeded in 48-well plates and cultured for 7 days on the extract of different samples and the Conditioned culture medium. FM was used as a control for monoculture, and CM was used as a control for the Co-culture model. The cells were incubated with primary antibodies: VE-cadherin Polyclonal Antibody (PA5-19612, Thermo Fisher Scientific, Invitrogen) and anti-CD31 antibody (ab24590, abcam) at 4˚C overnight, followed by a further incubation at RT for 1h with secondary antibodies, goat anti-rabbit IgG H & L (Alexa Fluor™ 488; ab150077, abcam) and goat anti-mouse IgG H & L (Alexa Fluor® 594; ab150116, abcam). Nuclear DNA was labeled in blue with DAPI. F-actin was stained with ActinGreen™ 488 ReadyProbe Reagent (AlexaFluor™ 488 phalloidin) and ActinRed™ 555 ReadyProbes reagent (Rhodamine phalloidin). Stained cells were imaged using a fluorescence microscope (Olympus I×83 microscope).

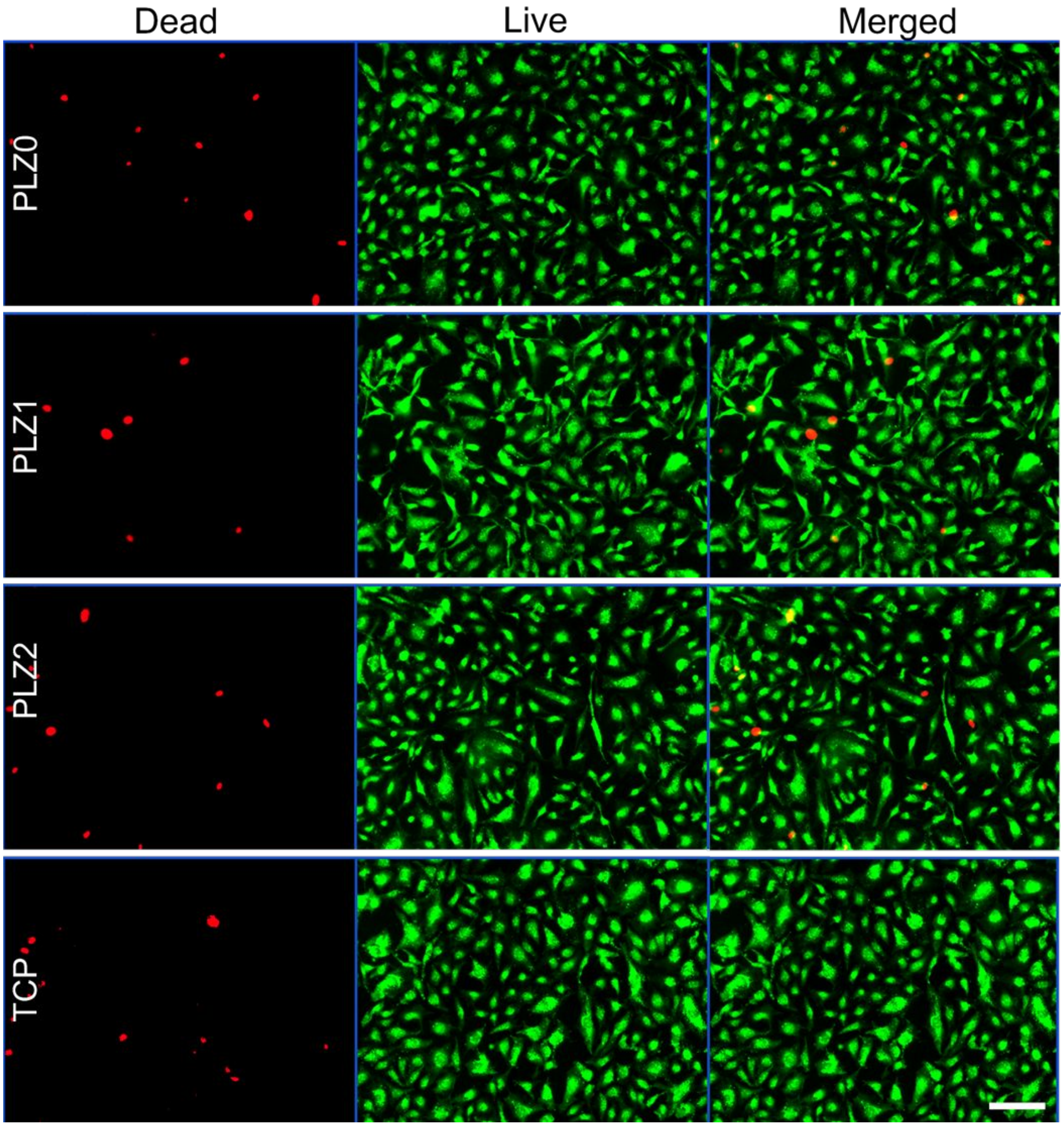

Figure S11. Fluorescence microscopy images of HUVECs cultured on Conditioned Medium, stained with live/dead staining dye at day 5 of the culture. Live cells (green color) and dead cells (red color) are stained using Perkin Elmer LLC Via Acridine Orange/ Propidium Iodide (AOPI) Staining Solution. Scale bar = 100µm.

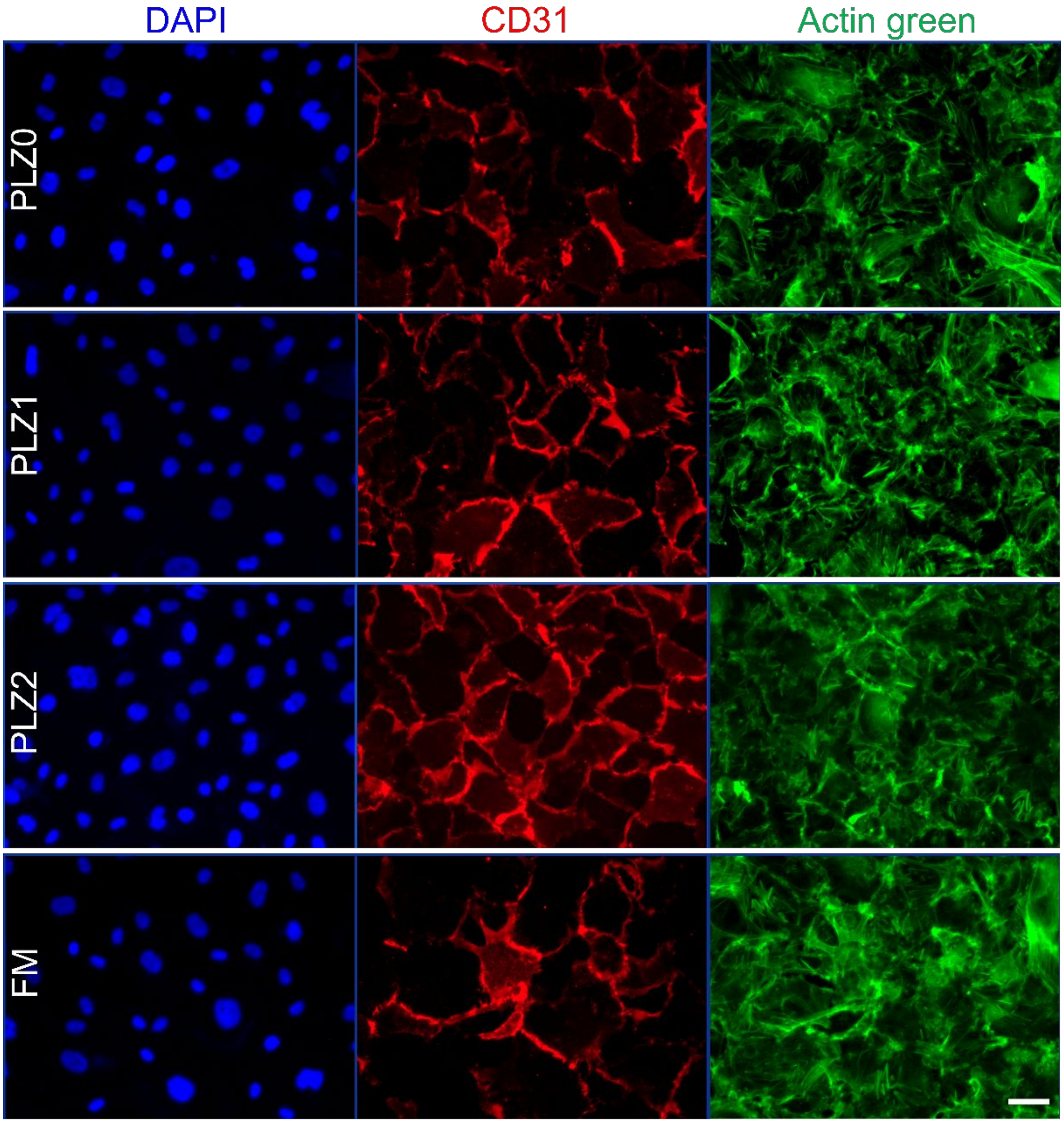

Figure S12. Immunofluorescence images displaying the endothelial-junction-associated protein markers, CD31 (red) by the HUVECs cultured under-extracted medium from different fibrous scaffolds for seven days. F-actin was stained with ActinGreen™ 488 ReadyProbes™ Reagents (AlexaFluor™ 488 phalloidin). Scale bar = 50 μm.

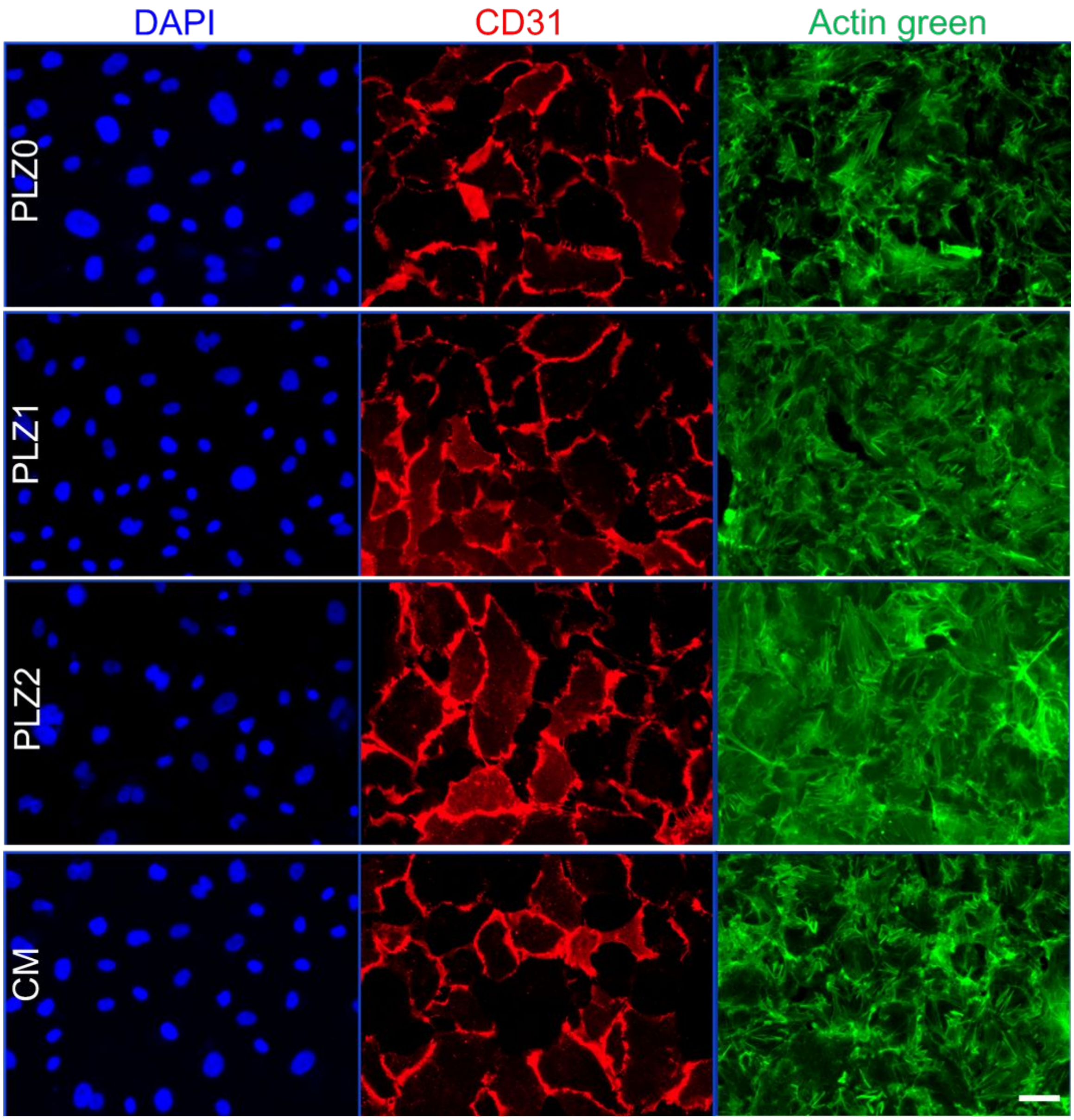

Figure S13. Immunofluorescence images displaying the endothelial-junction-associated protein markers CD31 (red) by the HUVECs cultured under Conditioned Medium from HDFn grown on different fibrous scaffolds for seven days. F-actin was stained with ActinGreen™ 488 ReadyProbes™ Reagents (AlexaFluor™ 488 phalloidin). Scale bar = 50 μm.

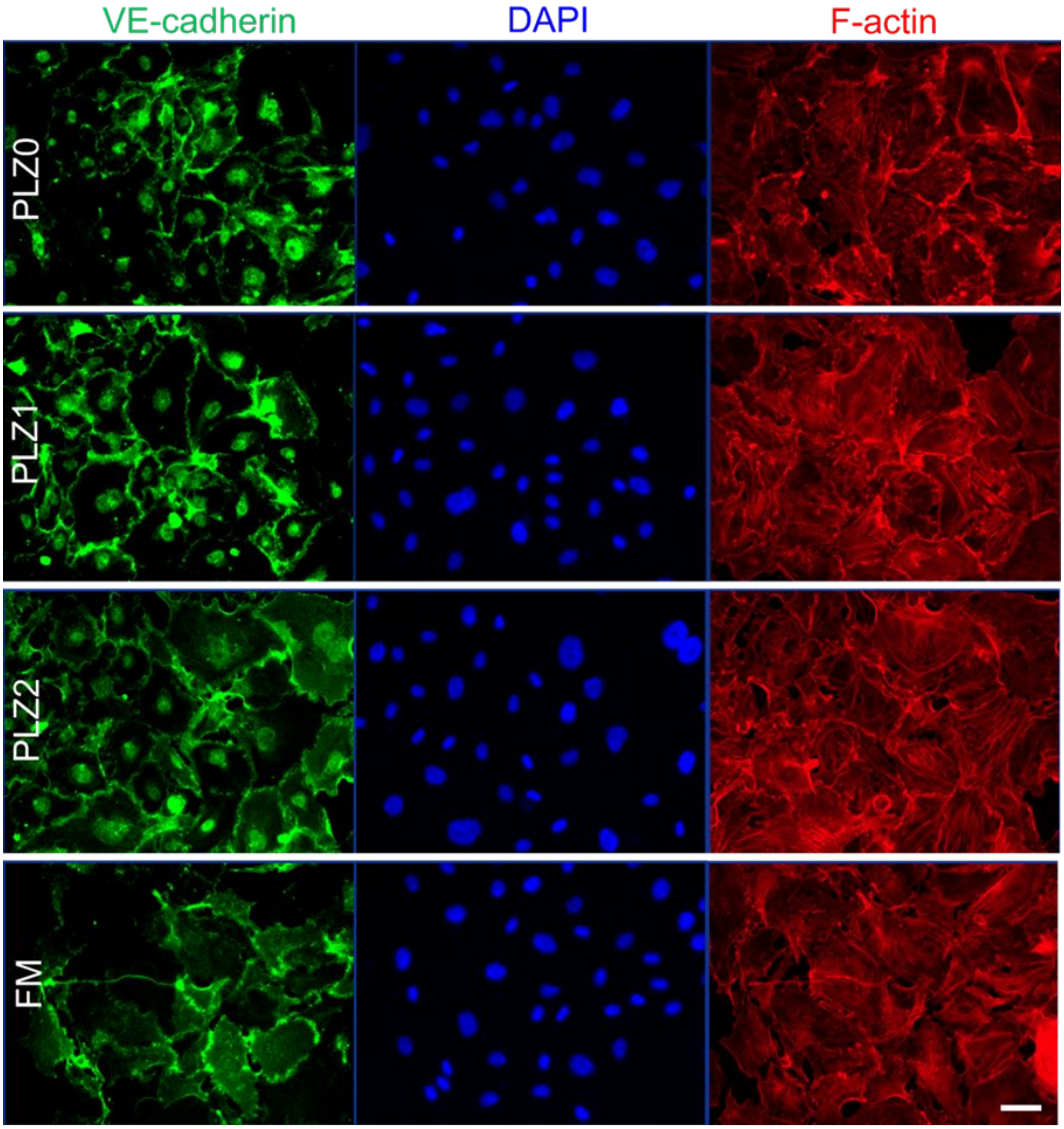

Figure S14. Immunofluorescence images displaying the endothelial-junction-associated protein markers, VE-cadherin (green), by the HUVECs cultured under-extracted medium from different fibrous scaffolds for seven days. F-actin was stained with ActinRed^{TM} 555 ReadyProbes^{TM} Reagents (Rhodamine phalloidin). Scale bar = 50 μm.

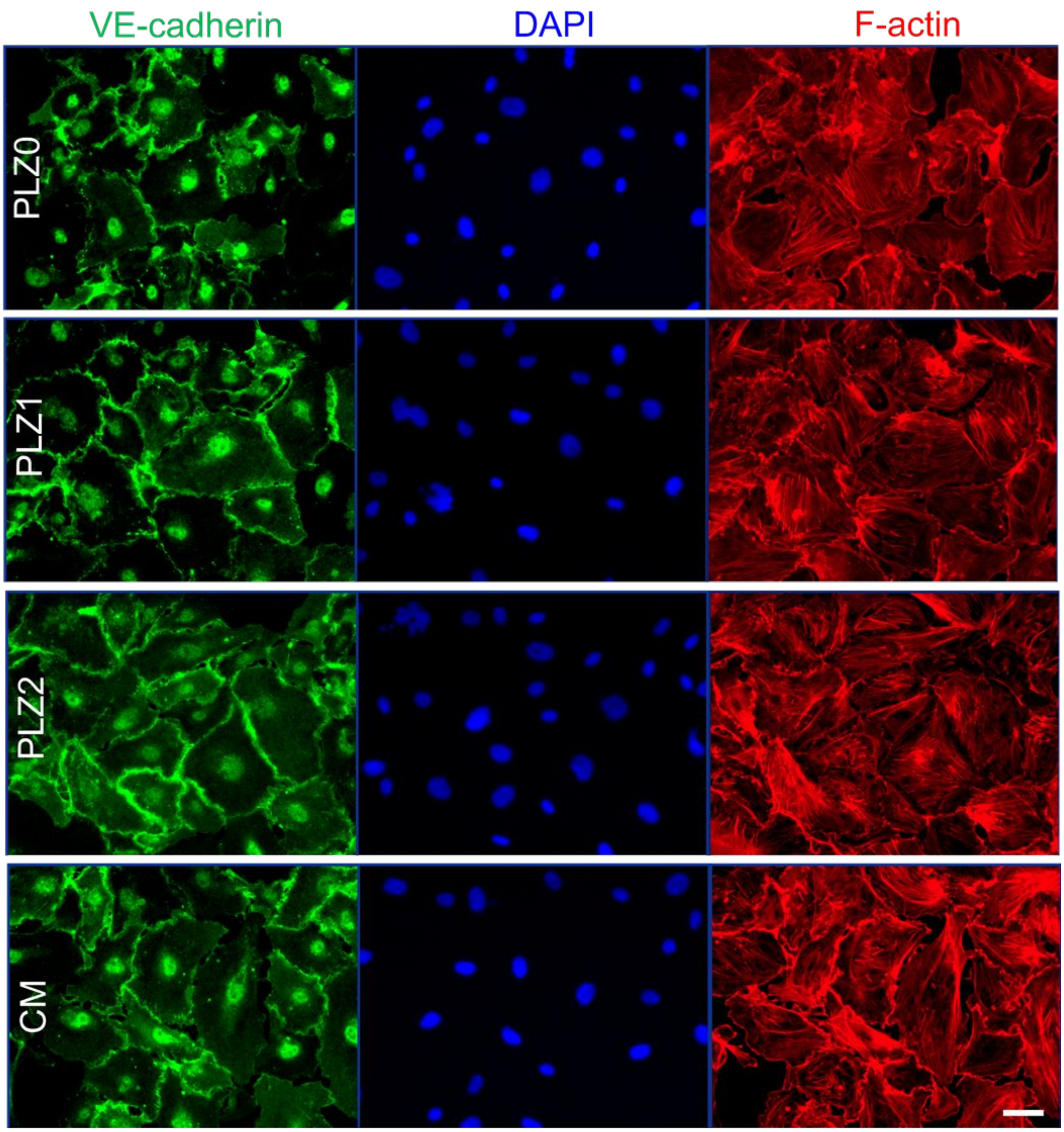

Figure S15. Immunofluorescence images displaying the endothelial-junction-associated protein, VE-cadherin (green), by the HUVECs cultured under Conditioned Medium from HDFn grown on different fibrous scaffolds for seven days. F-actin was stained with ActinRed^{TM} 555 ReadyProbes^{TM} Reagents (Rhodamine phalloidin). Scale bar = 50 μm.

References

(1) Yoshida, A.; Yamauchi, N.; Nakashima, K.; Watanabe, K.; Koda, H.; Kunigami, H.; Kunigami, H.; Kobayashi, Y. Synthesis of metallic zinc nanoparticles by electrolysis. Appl Nanosci 10: 3457–3464. **2020**.

(2) Mo, Y.; Guo, R.; Liu, J.; Lan, Y.; Zhang, Y.; Xue, W.; Zhang, Y. Preparation and properties of PLGA fibrous membranes reinforced with cellulose nanocrystals. *Colloids and Surfaces B: Biointerfaces* **2015***, 132*, 177–184.

(3) Lee, M.; Kim, T. G.; Kim, W.; Sung, Y. Surface plasmon resonance (SPR) electron and energy transfer in noble metal− zinc oxide composite nanocrystals. *The Journal of Physical Chemistry C* **2008***, 112*, 10079–10082.

(4) Blazsó, M. Thermal decomposition of polymers modified by catalytic effects of copper and iron chlorides. *J. Anal. Appl. Pyrolysis* **1999***, 51*, 73–88, DOI: 10.1016/S0165-2370(99)00009-1.

(5) Raam Dheep, G.; Sreekumar, A. Influence of nanomaterials on properties of latent heat solar thermal energy storage materials – A review. *Energy Conversion and Management* **2014***, 83*, 133–148, DOI: 10.1016/j.enconman.2014.03.058.

(6) Dejene, B. K. Advancing natural fiber-reinforced composites through incorporating ZnO nanofillers in the polymeric matrix: a review. *Journal of Natural Fibers* **2024***, 21*, 2356015.

(7) Feng, P.; Song, G.; Zhu, X.; Lv, D.; Zhao, Y.; Yang, X.; Li, N.; Zhang, L.; Ma, L. Enhanced interfacial adhesion of carbon fiber/epoxy composites by synergistic reinforcement with multiscale "rigid-flexible" structure at interphase. *Composites Part B: Engineering* **2021***, 225*, 109315.

(8) Yue, M.; Li, Y.; Hou, Y.; Cao, W.; Zhu, J.; Han, J.; Lu, Z.; Yang, M. Hydrogen bonding stabilized self-assembly of inorganic nanoparticles: mechanism and collective properties. *ACS nano* **2015***, 9*, 5807–5817.

(9) Shrestha, S.; Jang, S. R.; Shrestha, B. K.; Park, C. H.; Kim, C. S. Engineering 2D approaches fibrous platform incorporating turmeric and polyaniline nanoparticles to predict the expression of βIII-Tubulin and TREK-1 through qRT-PCR to detect neuronal differentiation of PC12 cells. *Materials Science and Engineering: C* **2021***, 127*, 112176.

(10) Cai, Q.; Shi, G.; Bei, J.; Wang, S. Enzymatic degradation behavior and mechanism of Poly(lactide-co-glycolide) foams by trypsin. *Biomaterials* **2003***, 24*, 629–638, DOI: 10.1016/S0142-9612(02)00377-0.
